# Supplementary material for: Engineering novel AAV capsids by broadly attenuated and subsequent muscle-specific tropism in mice and NHPs
Source: Mol Ther Adv. 2026 Mar 28;34(2):201725. doi: 10.1016/j.omta.2026.201725 (PMC13175773; doi:10.1016/j.omta.2026.201725)
Supplement: Document S1. Figures S1–S4 and Tables 7 and 8 [file mmc1.pdf]

OMTA, Volume *34*

Supplemental information

Engineering novel AAV capsids by broadly attenuated and subsequent muscle-specific tropism in mice and NHPs

Yue Pan, Yujian Zhong, Huan Chen, Youwei Zhang, Zhiyong Dai, Junlin Chen, Keqin Tan, Xiaoqu Chen, Danlan Qiu, Longxiang Sheng, Xinping Tan, Ying Fan, Ye Bu, Zexin Zhou, Zhiming Yang, Rui Duan, Min Guan, Guangping Gao, and Huapeng Li

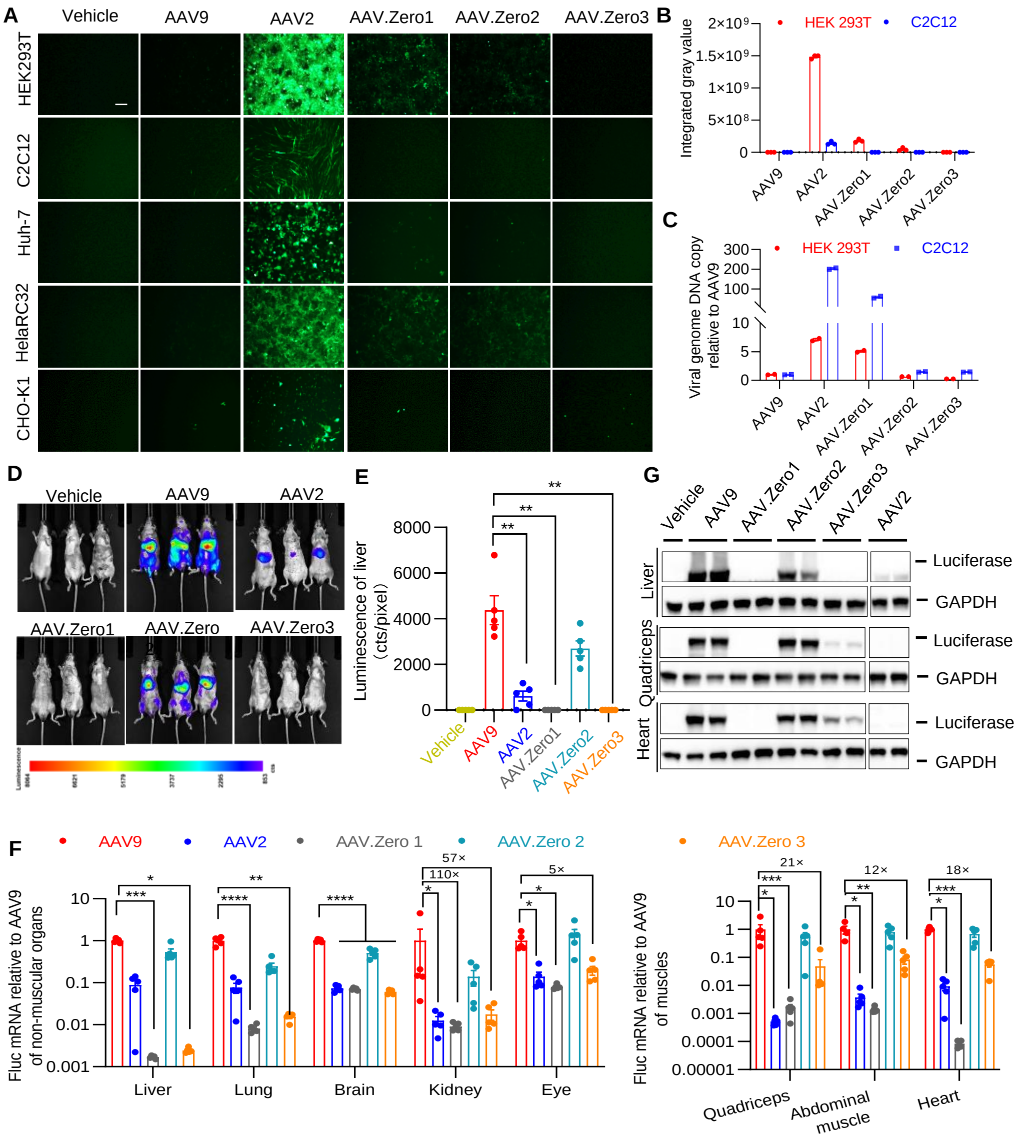
A
Vehicle
AAV9
AAV2
AAV.Zero1
AAV.Zero2
AAV.Zero3
HEK293T
C2C12
Huh-7
HelaRC32
CHO-K1
B
HEK 293T
C2C12
Integrated gray value
2×10⁹
1.5×10⁹
1×10⁹
5×10⁸
0
AAV9
AAV2
AAV.Zero1
AAV.Zero2
AAV.Zero3
C
HEK 293T
C2C12
Viral genome DNA copy relative to AAV9
300
200
100
10
5
0
AAV9
AAV2
AAV.Zero1
AAV.Zero2
AAV.Zero3
D
Vehicle
AAV9
AAV2
AAV.Zero1
AAV.Zero
AAV.Zero3
E
Luminescence of liver (cts/pixel)
8000
6000
4000
2000
0
**
**
**
Vehicle
AAV9
AAV2
AAV.Zero1
AAV.Zero2
AAV.Zero3
G
Vehicle
AAV9
AAV.Zero1
AAV.Zero2
AAV.Zero3
AAV2
Liver
Quadriceps
Heart
Luciferase
GAPDH
Luciferase
GAPDH
Luciferase
GAPDH
F
AAV9
AAV2
AAV.Zero 1
AAV.Zero 2
AAV.Zero 3
Fluc mRNA relative to AAV9 of non-muscular organs
10
1
0.1
0.01
0.001
*
***
**
****
****
57×
110×
*
5×
*
*
Liver
Lung
Brain
Kidney
Eye
Fluc mRNA relative to AAV9 of muscles
10
1
0.1
0.01
0.001
0.0001
0.00001
21×
***
*
12×
**
*
18×
***
*
Quadriceps
Abdominal muscle
Heart

Figure S1. The tropisms of chimeric capsid variants systematically decreased even at higher virus dose

A–C. HEK293T, C2C12, Huh-7, HelaRC32, and CHO-K1 cell lines being transduced with AAV9-, AAV2-, AAV.Zero1-, AAV.Zero2-, or AAV.Zero3-CAG-Fluc-P2A-EGFP at MOI=1E5 after 120 hours. A. Representative images of each treatment. Scale bar: 100 μm. B. Quantification of the integrated gray value of the EGFP fluorescence strength in HEK 293T and C2C12 cells (n=3). C. Quantification of relative virus genome copy numbers in HEK 293T and C2C12 cells (n=2). D–G. 8-week-old C57BL/6J mice were systemically injected with 1×10^{12} vg per mouse ($\sim 4 \times 10^{13}$ vg/kg) of AAV9-, AAV2-, AAV.Zero1-, AAV.Zero2-, or AAV.Zero3-CAG-Fluc-P2A-EGFP and data were collected 21 days post-injection. Representative whole body *in vivo* bioluminescence images (D). Quantification of firefly luciferase luminescence from liver (E) and quantification of fold-difference in *Fluc* mRNA expression in various tissues compared with normalized AAV9 (F). Comparisons between AAV9 and each of the other groups were performed using the Brown-Forsythe test and Welch's ANOVA, or the Kruskal-Wallis test, as appropriate for the data distribution and variance. (n=5 biologically independent animals, except for AAV9 abdominal muscle n=4), *$p < 0.05$, **$p < 0.01$, ***$p < 0.001$ and ****$p < 0.0001$. Representative western blot images detecting luciferase and GAPDH in quadriceps, heart, and liver (G).

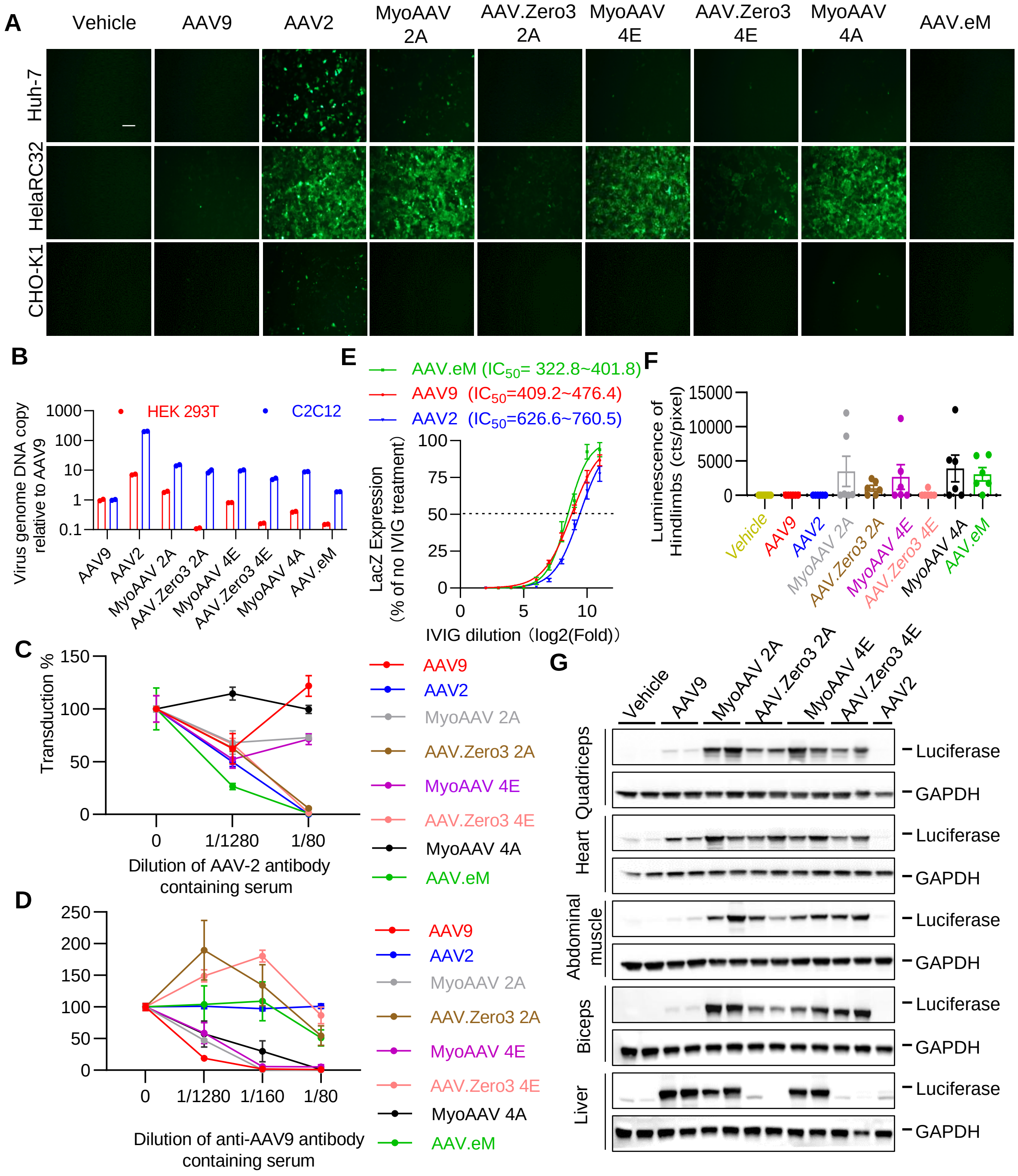
A
Vehicle
AAV9
AAV2
MyoAAV 2A
AAV.Zero3 2A
MyoAAV 4E
AAV.Zero3 4E
MyoAAV 4A
AAV.eM
Huh-7
HelaRC32
CHO-K1
B
Virus genome DNA copy relative to AAV9
HEK 293T
C2C12
E
AAV.eM (IC50= 322.8~401.8)
AAV9 (IC50=409.2~476.4)
AAV2 (IC50=626.6~760.5)
LacZ Expression (% of no IVIG treatment)
IVIG dilution（log2(Fold)）
F
Luminescence of Hindlimbs (cts/pixel)
C
Transduction %
Dilution of AAV-2 antibody containing serum
D
Dilution of anti-AAV9 antibody containing serum
G
Quadriceps
Heart
Abdominal muscle
Biceps
Liver
Luciferase
GAPDH

Figure S2. RGD-containing peptides enable chimeric capsid selectively target to muscle with high efficiency in C57BL/6J mice

A. Representative images Huh-7, HelaRC32, and CHO-K1 cell lines 72 hours after being transfected with AAV9-, AAV2-, MyoAAV 2A-, AAV.Zero3 2A-, MyoAAV 4E-, AAV.Zero3 4E-, MyoAAV 4A-, or AAV.eM-CAG-Fluc-P2A-EGFP at MOI=1E5. Scale bar: 100 μm. B. Quantification of relative virus genome copy numbers in HEK 293T and C2C12 cells transduced with AAV9-, AAV2-, MyoAAV 2A-, AAV.Zero3 2A-, MyoAAV 4E-, AAV.Zero3 4E-, MyoAAV 4A-, or AAV.eM-CAG-Fluc-P2A-EGFP at MOI=1E5 (n=2). C. Different dilutions of AAV2-antibody containing serum were incubated with constant amounts of virus at MOI=1E6 and tested for neutralization of transduction using an *in vitro* assay (n=4 for left panel and n=6 for right panel). D. Different dilutions of AAV9-antibody containing serum were incubated with constant amounts of virus at MOI=1E6 and tested for neutralization of transduction using an *in vitro* assay (n=3–6). E. Neutralization assay of AAV2, AAV9, MyoAAV 4A and AAV.eM using IVIG (n=4 wells), the confidence interval of IC_{50} is 95%. F-G. 8-week-old C57BL/6J mice were systemically injected with 2×10^{11} vg per mouse of AAV9-, AAV2-, MyoAAV 2A-, AAV.Zero3 2A-, MyoAAV 4E-, AAV.Zero3 4E-, MyoAAV 4A-, or AAV.eM-CAG-Fluc-P2A-EGFP- and data were collected 21 days post-injection. Quantification of the firefly luciferase luminescence intensity from hindlimbs (n=6 biologically independent animals) (F). Representative western blot images detecting luciferase and GAPDH in quadriceps, heart, abdominal muscle, biceps, and liver (G).

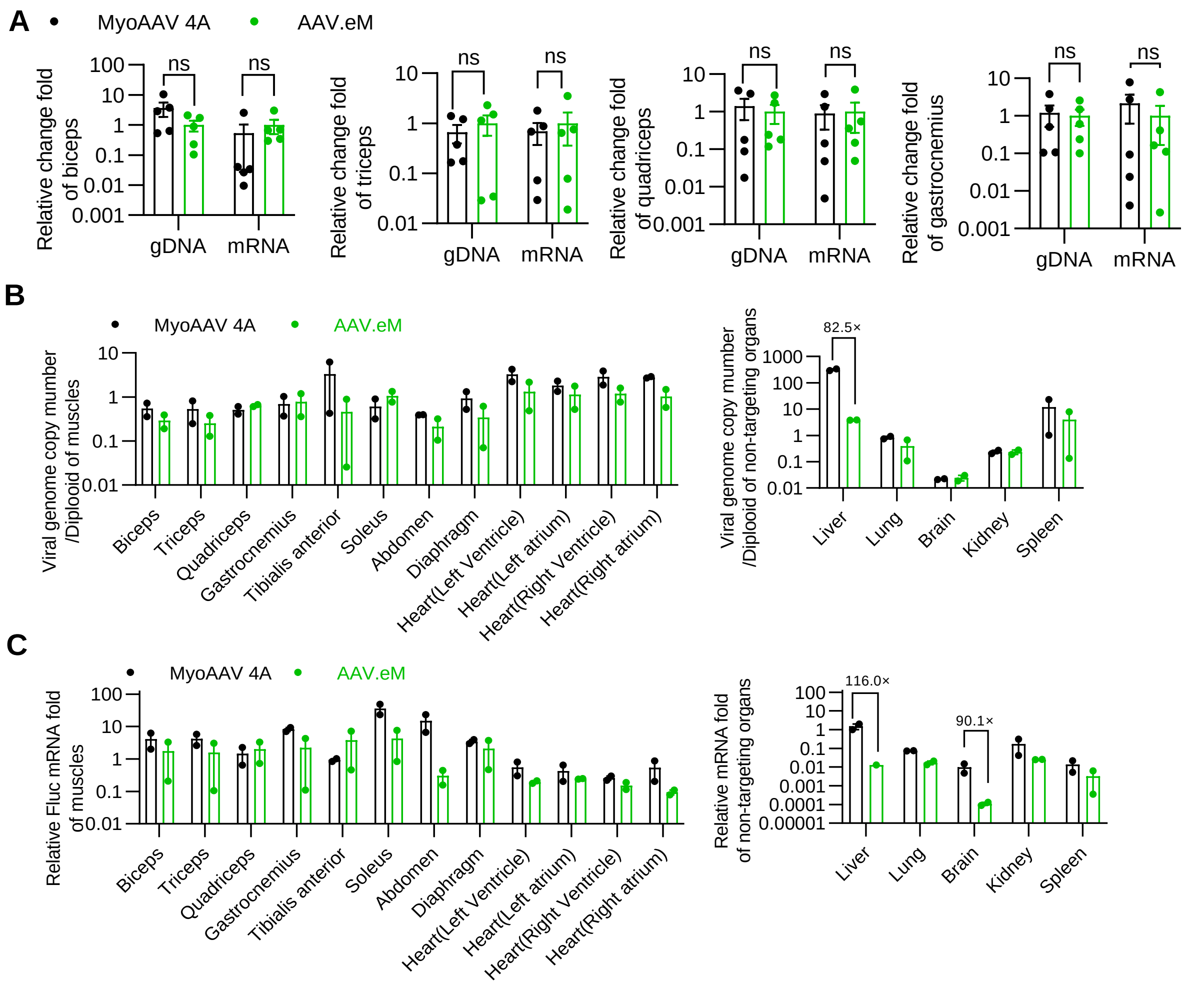
A
MyoAAV 4A
AAV.eM
Relative change fold of biceps
Relative change fold of triceps
Relative change fold of quadriceps
Relative change fold of gastrocnemius
ns
gDNA
mRNA
B
Viral genome copy number /Diploid of muscles
Biceps
Triceps
Quadriceps
Gastrocnemius
Tibialis anterior
Soleus
Abdomen
Diaphragm
Heart(Left Ventricle)
Heart(Left atrium)
Heart(Right Ventricle)
Heart(Right atrium)
Viral genome copy number /Diploid of non-targeting organs
82.5×
Liver
Lung
Brain
Kidney
Spleen
C
Relative Fluc mRNA fold of muscles
Relative mRNA fold of non-targeting organs
116.0×
90.1×

Figure S3. AAV.eM exhibits less off-target transduction and comparable muscle-specific targeting compared with MyoAAV 4A

A–C. NHPs were given intravenous administration of 3×10^{13} vg/kg of MyoAAV 4A- or AAV.eM-CAG-Fluc-P2A-EGFP. Relative change fold of viral gDNA copy and *Fluc* mRNA in biceps, triceps, quadriceps and gastrocnemius sampled 28 days after virus injection (A). Comparisons between two groups were performed using Student's *t*-test, Welch's *t*-test, or the Mann-Whitney U test, as appropriate for the data distribution and variance. (n=5 biologically independent animals), ns: not significant. The biceps, triceps, quadriceps, gastrocnemius, tibialis anterior, soleus, abdominal muscle, diaphragm, heart, liver, lung, brain, kidney, and spleen were sampled 28 days after virus injection (n=2 biologically independent animals). Absolute quantification of viral vector genomes per diploid of each tissue was assayed by ddPCR (B). Fold-change in *Fluc* mRNA expression relative to *GAPDH* as assayed by qPCR with standard curve (C).

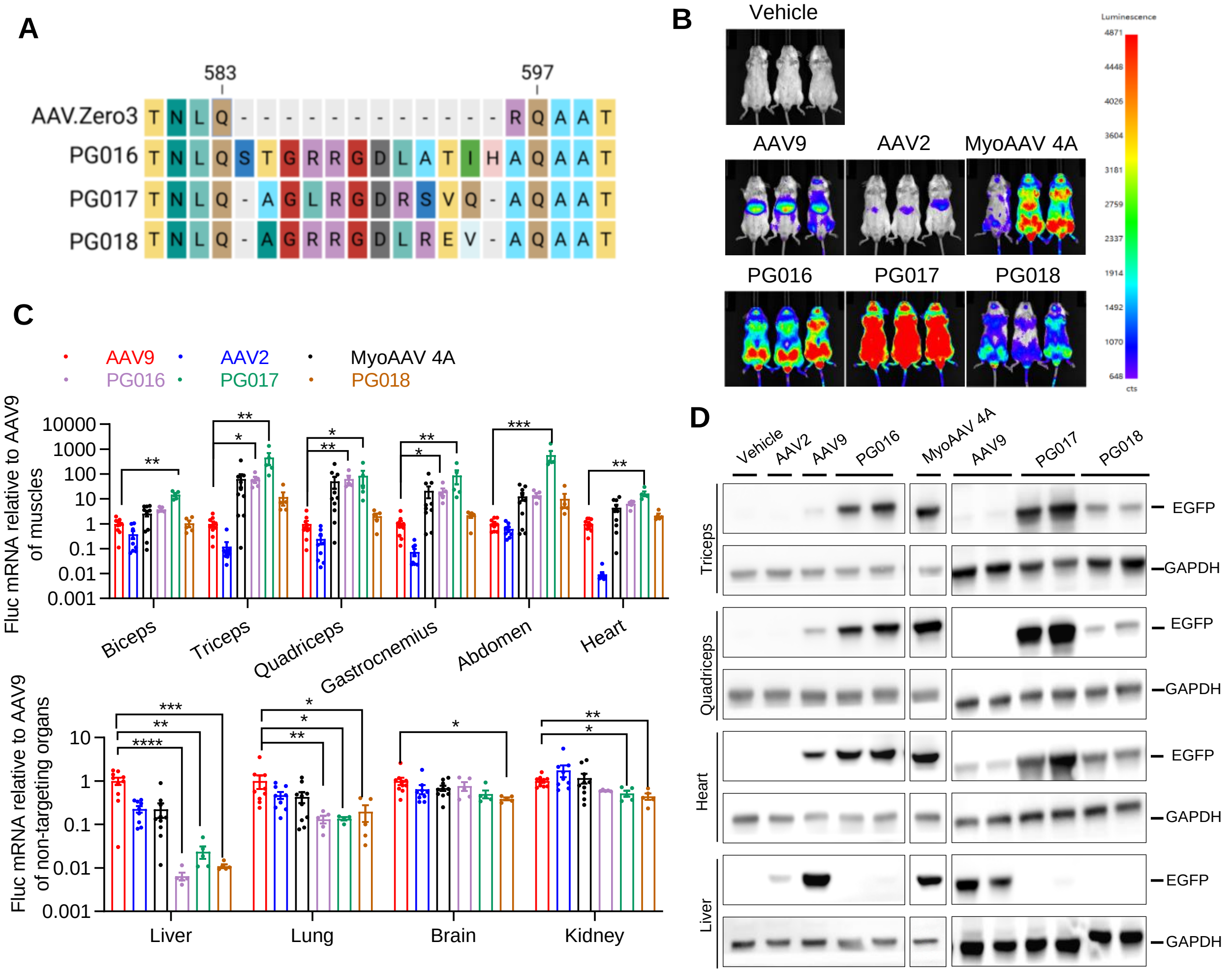
A
583
597
AAV.Zero3 T N L Q - - - - - - - - - - - - R Q A A T
PG016 T N L Q S T G R R G D L A T I H A Q A A T
PG017 T N L Q - A G L R G D R S V Q - A Q A A T
PG018 T N L Q - A G R R G D L R E V - A Q A A T
B
Vehicle
AAV9
AAV2
MyoAAV 4A
PG016
PG017
PG018
Luminescence
4871
4448
4026
3604
3181
2759
2337
1914
1492
1070
648
cts
C
AAV9
AAV2
MyoAAV 4A
PG016
PG017
PG018
Fluc mRNA relative to AAV9 of muscles
10000
1000
100
10
1
0.1
0.01
0.001
Biceps
Triceps
Quadriceps
Gastrocnemius
Abdomen
Heart
Fluc mRNA relative to AAV9 of non-targeting organs
10
1
0.1
0.01
0.001
Liver
Lung
Brain
Kidney
D
Vehicle
AAV2
AAV9
PG016
MyoAAV 4A
AAV9
PG017
PG018
Triceps
Quadriceps
Heart
Liver
EGFP
GAPDH

Figure S4. Novel muscle targeting peptides based on low-background capsid exhibit selectively target to muscles with high efficiency

A–D. 8-week-old BALB/c mice were systemically injected with 2×10^{11} vg/mouse of AAV9-, AAV2-, MyoAAV 4A, PG016, PG017, or PG018-CAG-Fluc-P2A-EGFP and tissues were collected 21 days post-injection. Sequence alignments of the RGD-containing peptide insertion region of PG016, PG017, and PG018 compared with AAV.Zero3 (A). Representative whole body *in vivo* bioluminescence images (B), the images for the Vehicle, AAV9, and AAV2 control groups are the same as those presented in Figure 3A. These panels are derived from the same experimental batch and are reused here as a common internal control to enable a direct and consistent comparison with the novel capsids under investigation. Quantification of fold-difference in *Fluc* mRNA expression in various tissues (C) compared with normalized AAV9. Comparisons between AAV9 and each other group were performed using Kruskal-Wallis tests. Sample sizes were n = 10 for common internal control groups and n = 5 for test groups (PG016–PG018), with the following exceptions due to a single sample lacking a detectable signal: AAV2 in lung (n = 9) and PG017 in lung (n = 4). Each sample represents a biologically independent animal. Representative western blot images detecting EGFP and GAPDH in varied tissues (D).

Table S1. Experimental design for barcode-mediated sorting of AAV variants in NHPs.

Three monkeys received an intravenous co-injection of five AAV variants (MyoAAV 4A, PG016, PG017, PG018, AAV9) at a total dose of 3E13 GC/kg, equally distributed. Muscle (gastrocnemius, biceps, triceps, quadriceps) and liver punctures were performed at Day 14 and Day 28. Each muscle puncture sample was divided into three parts for analysis. Viral tropism was assessed via NGS barcode sequencing of the collected tissues. Data include raw DNA counts, calculated relative fold-enrichment versus AAV9, and summary statistics (mean ± STD) across biological replicates for each sample at Day 14 and Day 28.

Table S2. NGS barcode sequencing counts and relative tissue tropism analysis for the five AAV variants in the liver of NHPs.

Table S3. NGS barcode sequencing counts and relative tissue tropism analysis for the five AAV variants in the gastrocnemius of NHPs.

Table S4. NGS barcode sequencing counts and relative tissue tropism analysis for the five AAV variants in the biceps of NHPs.

Table S5. NGS barcode sequencing counts and relative tissue tropism analysis for the five AAV variants in the triceps of NHPs.

Table S6. NGS barcode sequencing counts and relative tissue tropism analysis for the five AAV variants in the quadriceps of NHPs.

Table S7. AAV.eM demonstrates superior stability and reduced aggregation compared to MyoAAV 4A in AAV production.

Batch Scale (L)	Gene of Interest （GOI）	Capsid	Lysate Genome Titer (vg/mL)	DP Genome Titer (vg/mL)	Appearance	Z-average Diameter (nm)	PDI	SD (nm)
200	GOI-1	MyoAAV 4A	4.79E+11	1.08E+13	Colorless Transparent	785.66	0.383	30.85
200	GOI-2	MyoAAV 4A	5.02E+11	1.41E+13	Opalescent Cloudy	1185.68	0.127	422.99
				8.70E+12	Colorless Transparent	28.12	0.077	7.8
				4.34E+12	Colorless Transparent	27.11	0.062	6.76
3	GOI-3	AAV.eM	2.16E+11	3.01E+13	Colorless Transparent	30.33	0.062	7.56
3	GOI-4	AAV.eM	3.22E+11	5.11E+13	Colorless Transparent	32.6	0.108	10.69
3	GOI-5	AAV.eM	3.66E+11	1.18E+14	Colorless Transparent	29.37	0.047	6.37

Note： DP, drug product; PDI, polydispersity index; SD, standard deviation.

Table S8. Primers

Primer	Sequence
**Backbone construction**	
Cap-F	CATCTTTGAACAATAAATGATTTAAATCAGGTATG
Cap-R	TCAACTGAAACGAATCAACCGGTTT
YJ69-R	TGTTGATTCTGTCCAGAACCGTTAATAGTCTTGCTCAAGTAATACAGGTACTG GTCGAT
YJ69-F	ACGGTTCTGGACAGAATCAACAAACGCTAAAATTTTCTCAGGCCGGAGCGAG T
YJ72-R	GTTGTTTTGAGTCACAGTGGTTGATACTCGCTGCTGGCGGTAACAG
YJ72-F	ATCAACCACTGTGACTCAAAACAACAACAGTGAATACTCGTGGACTGGAG
247-R	TTGTCTGTTGCCAGCCTGGAGGTTGGTAGATACAGAACCATACT
247-F	CCTCCAGGCTGGCAACAGACAAGCAGCTACCGCAGAT
248-R	CTGCTTGTCTCTGGAGGTTGGTAGATACAGAACCATACT
248-F	CAACCTCCAGAGACAAGCAGCTACCGCAGAT
**Peptide insertion**	
249-R	TGTTGTCTGGTCTCCTCTTCCAGGTCCCTGGAGGTTGGTAGATACAGAACCAT ACT
249-F	CCTGGAAGAGGAGACCAGACAACACTGAGACAAGCAGCTACCGCAGAT
250-R	ATTGTTGAAATCGCCTCGTCTATTCTCCTGGAGGTTGGTAGATACAGAACCAT ACT
250-F	AATAGACGAGGCGATTTCAACAATACCAGACAAGCAGCTACCGCAGAT
YJ107-R	GAGTTGTAGTCTCCTCTGCTGTTGCTCTGGAGGTTGGTAGATACAGAACCATA CT
YJ107-F	GCAACAGCAGAGGAGACTACAACTCCCTGAGACAAGCAGCTACCGCAGAT
**Viral titer**	
ITR-F	GGAACCCCTAGTGATGGAGTT
ITR-R	CGGCCTCAGTGAGCGA
**qPCR**	
Luciferase	F-AACCAGCGCCATTCTGATCA; R-TCGGGGTTGTTAACGTAGCC
GAPDH (mouse)	F-CAGGAGAGTGTTTCCTCGTCC; R-TTCCCATTCTCGGCCTTGAC
GAPDH (monkey)	F-GTCTCCTCTGACTTCAACAGCG; R-ACCACCCTGTTGCTGTAGCCAA
**ddPCR**	
Luciferase	F-GCTTCCACCTACCAGGCAT; R-TCTTACCGGTGTCCAAGTCC
	Probe-CCGCCAGGGCTACGGCCTGA
GAPDH (mouse)	F-GATGTCCTTGGTGCACACTC; R-GAGCTGAGATTGCCCCGC
	Probe-CCTCGTCCTTAAGTTCATAGTCTGTATTC
GAPDH (monkey)	F-CGGGTCTTTGCTGTCGTATG; R-CTGCACAGGGCGCCACAGCC
	Probe-GAGTAGGGACCTCCTGTCCT
