# Supplementary material for: Engineering novel AAV capsids by broadly attenuated and subsequent muscle-specific tropism in mice and NHPs
Source: Mol Ther Adv. 2026 Mar 28;34(2):201725. doi: 10.1016/j.omta.2026.201725 (PMC13175773; doi:10.1016/j.omta.2026.201725)
Supplement: Document S2. Article plus supplemental information [file mmc3.pdf]

Molecular Therapy
Advances
Original Article

# Engineering novel AAV capsids by broadly attenuated and subsequent muscle-specific tropism in mice and NHPs

Yue Pan,[1,5] Yujian Zhong,[1,5] Huan Chen,[1] Youwei Zhang,[1] Zhiyong Dai,[1] Junlin Chen,[1] Keqin Tan,[1] Xiaoqu Chen,[1] Danlan Qiu,[1] Longxiang Sheng,[1] Xinping Tan,[1] Ying Fan,[1] Ye Bu,[1] Zexin Zhou,[1] Zhiming Yang,[1] Rui Duan,[2] Min Guan,[3] Guangping Gao,[4] and Huapeng Li[1]

[1]PackGene Biotech Inc, Houston, TX 77054, USA; [2]Laboratory of Regenerative Medicine in Sports Science, School of Physical Education and Sports Science, South China Normal University, Guangzhou 510006, China; [3]Research Center for Human Tissues and Organs Degeneration, Institute of Biomedicine and Biotechnology, Shenzhen Institute of Advanced Technology, Chinese Academy of Sciences, Shenzhen, China; [4]Department of Genetic and Cellular Medicine, Horae Gene Therapy Center, Li Weibo Institute for Rare Diseases Research, and Department of Microbiology, University of Massachusetts Chan Medical School, Worcester, MA 01605, USA

**Recombinant adeno-associated virus (rAAV) vectors are a potent gene delivery tool, but their clinical application is restricted by poor transduction of target tissues and off-target toxicity. To address this, we employed a two-step capsid engineering strategy: broad attenuation of rAAV tropism followed by peptide-driven tissue-specific retargeting. We first generated AAV.Zero1, a capsid with markedly reduced transduction across tissues, by VR swapping from AAV9 into AAV2. Introducing an R585A substitution (AAV.Zero2) partially restored the transduction, while deletion of residues 585–587 (AAV.Zero3) abolished it. By inserting a myogenic peptide into the AAV.Zero3 backbone, we produced a novel capsid (AAV.eM), which drove robust muscle-specific transgene expression with minimal off-target transduction in the liver, lung, brain, and kidney. This favorable profile was consistent across two mouse strains and non-human primates. AAV.eM mediated expression levels comparable to the leading myotropic vector, MyoAAV 4A, but exhibited a superior safety profile. Importantly, AAV.eM was able to functionally rescue a mouse model of Duchenne muscular dystrophy following systemic delivery of a micro-dystrophin gene. These results establish AAV.eM as an improved myotropic vector with enhanced specificity and proof of concept for a platform to create capsids with specific properties that translate across species by addition of peptides onto low transduction backbones.**

## INTRODUCTION

Recombinant adeno-associated virus (rAAV) vectors are the most commonly used viral platforms for gene delivery.[1–4] However, the clinical application faces two major limitations: systemic administration often leads to significant sequestration of viral capsids in the liver, while transduction efficiency in target organs and cells remains insufficient for therapeutic benefit.[5] Achieving optimal outcomes in muscle-related diseases typically requires high viral doses (approximately 1–3 $\times$ $10^{14}$ vg/kg), which presents challenges for virus production as well as concerns for off-target toxicity, and may provoke host anti-adeno-associated virus (AAV) immune responses.[6,7] Several clinical trials have reported patient fatalities following high-dose intravenous (i.v.) AAV administration, with severe adverse events including liver, kidney, heart, and lung failures.[8–11] Additionally, AAV-induced dorsal root ganglion (DRG) toxicity has been observed across multiple animal models, including non-human primates (NHPs), piglets, rabbits, and rats.[12,13] High-titer AAV can also lead to localized or systemic neurotoxicity within the central and peripheral nervous systems.[14,15] Consequently, de-targeting AAV capsids from non-therapeutic tissues has been an area of active interest in the field of gene therapy.[16,17]

Capsid engineering to optimize tropism for specific cells and tissues is a promising strategy for enhancing the clinical efficacy of AAV vectors. By increasing targeting precision, this approach can reduce the viral dose required to achieve therapeutic effects,[18,19] which is a key strategy for reducing overall immunogenicity and improving the

Received 29 September 2025; accepted 25 March 2026;
https://doi.org/10.1016/j.omta.2026.201725.

[5]These authors contributed equally

**Correspondence:** Rui Duan, Laboratory of Regenerative Medicine in Sports Science, School of Physical Education and Sports Science, South China Normal University, Guangzhou 510006, China.
**E-mail:** duanrui@m.scnu.edu.cn
**Correspondence:** Min Guan, Research Center for Human Tissues and Organs Degeneration, Institute of Biomedicine and Biotechnology, Shenzhen Institute of Advanced Technology, Chinese Academy of Sciences, Shenzhen, China.
**E-mail:** min.guan@siat.ac.cn
**Correspondence:** Guangping Gao, Department of Genetic and Cellular Medicine, Horae Gene Therapy Center, Li Weibo Institute for Rare Diseases Research, and Department of Microbiology, University of Massachusetts Chan Medical School, Worcester, MA 01605, USA.
**E-mail:** guangping.gao@umassmed.edu
**Correspondence:** Huapeng Li, PackGene Biotech Inc, Houston, TX 77054, USA.
**E-mail:** paul.li@packgene.com


Published by Elsevier Inc. on behalf of The American Society of Gene and Cell Therapy.
This is an open access article under the CC BY license (http://creativecommons.org/licenses/by/4.0/).

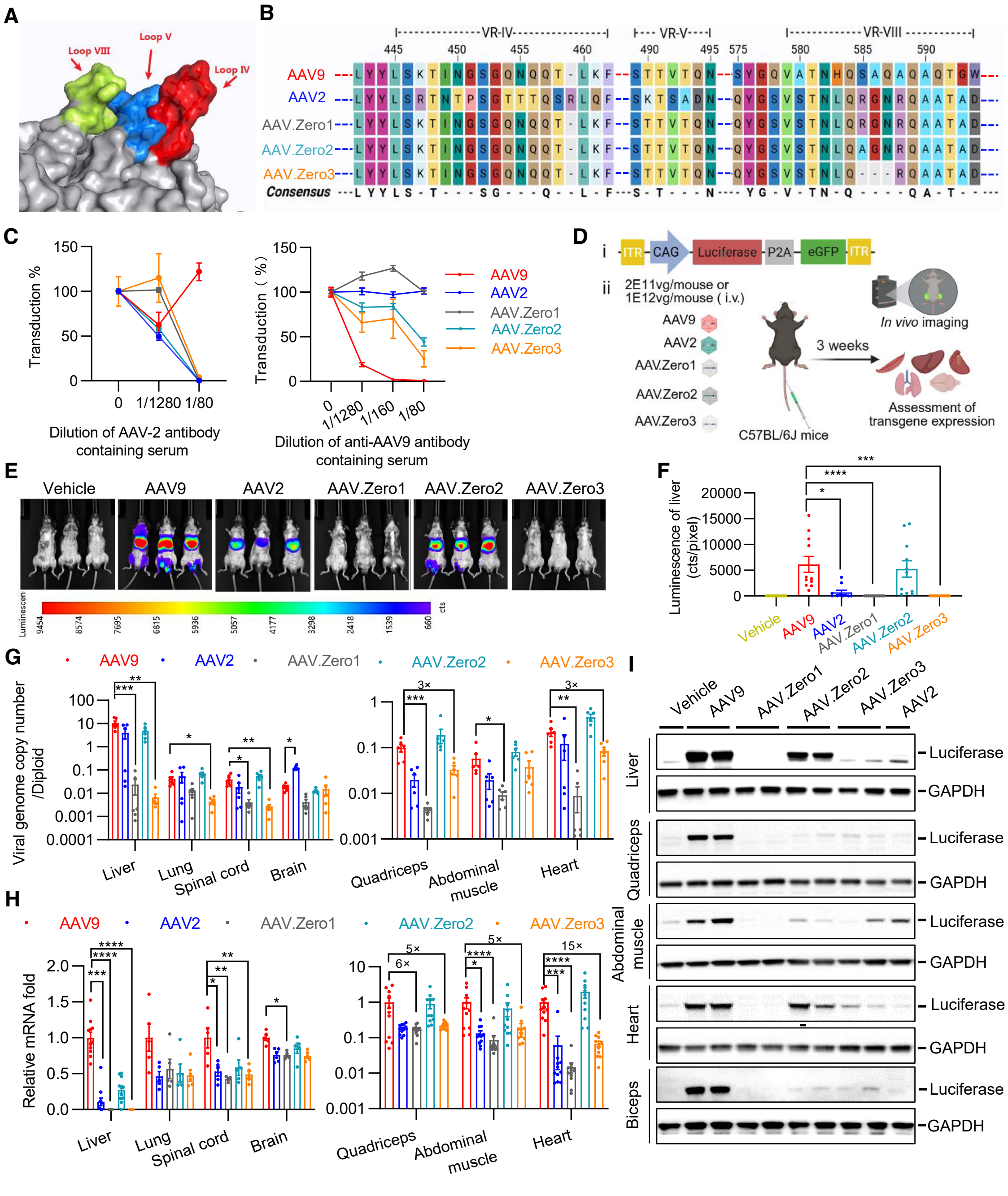


**Figure 1. Chimeric capsid variants exhibit dramatic reductions in liver transduction**

(A) Capsid spike formed by VRs IV and VIII of one AAV2 monomer interacting with the VR V of a second monomer. (B) Sequence alignments of the variable region (VR)-IV, VR-V, and VR-VIII of AAV.Zero1, AAV.Zero2, and AAV.Zero3 compared with AAV2 and AAV9. The blue dashed line represents the AAV2 backbone, while the red dashed line indicates the AAV9 backbone. (C) Different dilutions of AAV2 (left) or AAV9 (right) antibody-containing serum were incubated with constant amounts of AAV serotypes AAV9-, AAV2-, AAV.Zero1-, AAV.Zero2-, or AAV.Zero3-CAG-Fluc-P2A-EGFP at MOI = 1E6 and tested for neutralization of transduction in HEK293T. Data are presented as mean ± SEM ($n$ = 4 wells for left image, and $n$ = 6 wells for right image). (D) Schematic of virus administration and biodistribution assessment process. (1) CAG-luciferase-P2A-eGFP was packaged as reporter transgene. (2) Experimental workflow in mice.

(E–I) 8-week-old C57BL/6 J mice systemically injected with $2 \times 10^{11}$ vg per mouse (~$8 \times 10^{12}$ vg/kg) of AAV9-, AAV2-, AAV.Zero1-, AAV.Zero2-, or AAV.Zero3-CAG-Fluc-P2A-EGFP, and data were collected 21 days post-injection. Representative whole body *in vivo* bioluminescence (E) and quantification of firefly luciferase luminescence (F). A Kruskal-Wallis test was conducted, followed by Dunn's post hoc tests comparing AAV9 against each of the other capsid variants. Sample sizes consisted of 11 mice per group, with the exception of the AAV2 group, which comprised 10 biologically independent animals due to the loss of one mouse prior to imaging. *$p < 0.05$, ***$p < 0.001$,

*(legend continued on next page)*

safety profile of AAV therapies.[20] AAV tropism is governed by receptor-binding motifs on the capsid surface, which mediate viral attachment to cells and facilitate entry. Therefore, deciphering the structure-function relationship of these motifs—as well as the dynamic processes following attachment, such as internalization, trafficking, endosomal escape, nuclear import, uncoating, and genome processing—could enable the design of AAV variants with finely tailored tropisms.[21]

Effective viral transduction requires transgene expression, making the detection of capsids or vector genomes insufficient for confirming successful transduction. This has spurred the development of transcription-based screening platforms such as Tracer and Deliver for novel capsid engineering.[22,23] Although bioinformatics tools aid capsid design, *in vivo* validation remains necessary to identify potent and target tissue-enhanced capsids. A major translational challenge is the frequent failure of capsids optimized in murine models to maintain efficacy in NHPs, highlighting significant inter-species difference in AAV transduction.[24–28]

In this study, we developed three novel capsids based on rational, structure-guided design. AAV.Zero1 and AAV.Zero3 exhibit broadly low transduction across diverse mouse tissues, serving as universal backbones for targeted tropism via peptide insertion. As a proof of principle, engineering AAV.Zero3 with a muscle-targeting peptide produced AAV.eM—a capsid that enables highly efficient muscle-specific gene transfer with minimal non-therapeutic tissue transduction in both rodents and NHPs following systemic delivery. This approach promises to enhance the efficiency of capsid development, yielding more specific and safer vectors for gene therapy. It will enable the use of lower viral doses, streamline manufacturing for preclinical and clinical applications, and accelerate the development of effective AAV gene therapies for a wide range of human diseases.

## RESULTS

### Chimeric capsid variants exhibit dramatically lower background tropism in mice

Numerous AAV serotypes, including natural variants AAV7, AAV8, and AAV9 as well as novel engineered variants, exhibit high liver tropism following i.v. administration.[1,2] To mitigate hepatic and systemic toxicities, we sought to engineer capsids that de-target the liver by modifying motifs of the AAV2 capsid, which has reduced liver tropism compared to AAV9. The surface-exposed variable region (VR)-IV is the outermost protrusion along the viral capsid's 3-fold symmetry axis. Previous studies indicate that receptor-binding interactions occur near this axis, where VRs IV and VIII form adjacent surface VRs within a single monomer and further interact with VR-V from a neighboring monomer in AAV9.[18] In AAV2 (PDB: 6IH9), the positions of VRs IV, V, and VIII are similarly positioned in close proximity (Figure 1A). VR-IV has been reported to be critical for the efficient liver transduction of AAV8[29] and has previously been implicated in neutralizing antibody binding.[30] We hypothesized that these VRs participate in a structure-function relationship governing cell interactions and contribute to structural context-dependent tissue tropisms. To test this, we replaced two VRs in AAV2 (VR-IV: R447-Q461; VR-V: K490-D494) with the corresponding sequences from AAV9 (VR-IV: K449-K462; VR-V: T491-Q495), generating the chimeric capsid AAV.Zero1. Furthermore, since residue R585 in AAV2 is known to mediate binding to heparan sulfate proteoglycan (HSPG)—a key receptor for hepatotropism,[31] we introduced additional mutations into AAV.Zero1 to further diminish liver tropism. This resulted in two new variants: AAV.Zero2 (R585A) and AAV.Zero3 (R585GN deletion) (Figure 1B).

We assessed the transduction profiles of these three new capsid variants by assessing their efficiency in transducing five immortalized cell lines from different tissues, kidney HEK293T, myoblast C2C12, hepatoma Huh-7, cervical cancer cell HeLaRC32, and Chinese hamster ovary (CHO-K1). Each variant exhibited a distinct tropism profile compared to wild-type AAV2 (Figure S1A). Transgene delivery efficiency was evaluated via EGFP expression, quantified by fluorescence imaging in HEK293T and C2C12 cells (Figure S1B). Cellular entry efficiency was assessed by measuring relative vector genome copy number across cell types (Figure S1C), which generally correlated with observed EGFP expression levels (cf. Figures S1B and S1C). The presence of pre-existing anti-AAV antibodies in humans poses a major hurdle for *in vivo* gene therapy, as the antibodies can neutralize AAV-based gene delivery vectors.[32,33] Therefore, we evaluated the cross-reactivity of anti-AAV2 and anti-AAV9 antibodies against the three newly developed capsids using a neutralization inhibition assay. These novel vectors are derived from the AAV2 serotype and the results indicate that they cross-reacted with antibodies produced against AAV2 (Figure 1C).

To investigate the transduction profile and biodistribution of the chimeric variants compared to the parental capsids, we intravenously administered AAV9-, AAV2-, AAV.Zero1-, AAV.Zero2-, and AAV.Zero3-CAG-luciferase-P2A-EGFP to adult C57BL/6J mice at dosages of 2E11 or 1E12 viral genomes (vgs) per mouse (Figures 1D–1I and S1D–S1G). *In vivo* bioluminescence imaging revealed that AAV.Zero1 and AAV.Zero3 had significantly reduced

****$p < 0.0001$. (G) Viral genome copy number per diploid from mice tissues detected by droplet digital PCR. For each tissue, comparisons between AAV9 and the other capsid variants were made using a Kruskal-Wallis test, with significant results followed by Dunn's post hoc tests. ($n$ = 6 biologically independent mice for each group), *$p < 0.05$, **$p < 0.01$, ***$p < 0.001$. (H) Quantification of fold difference in *Fluc* mRNA expression. For each tissue, the appropriate statistical test was chosen to compare AAV9 with each other capsid variant: Welch's ANOVA (Brown-Forsythe test) for normally distributed data with unequal variances, or the Kruskal-Wallis test for non-normally distributed data, followed by Dunnett's T3 multiple comparisons test. The number of mice were 10–11 for each group of quadriceps, abdominal muscle and heart, and 4–5 for lung, spinal cord, and brain), *$p < 0.05$, **$p < 0.01$, ***$p < 0.001$, ****$p < 0.0001$. (I) Representative western blot images detecting luciferase and GAPDH of liver, quadriceps, abdominal muscle, heart, and biceps.

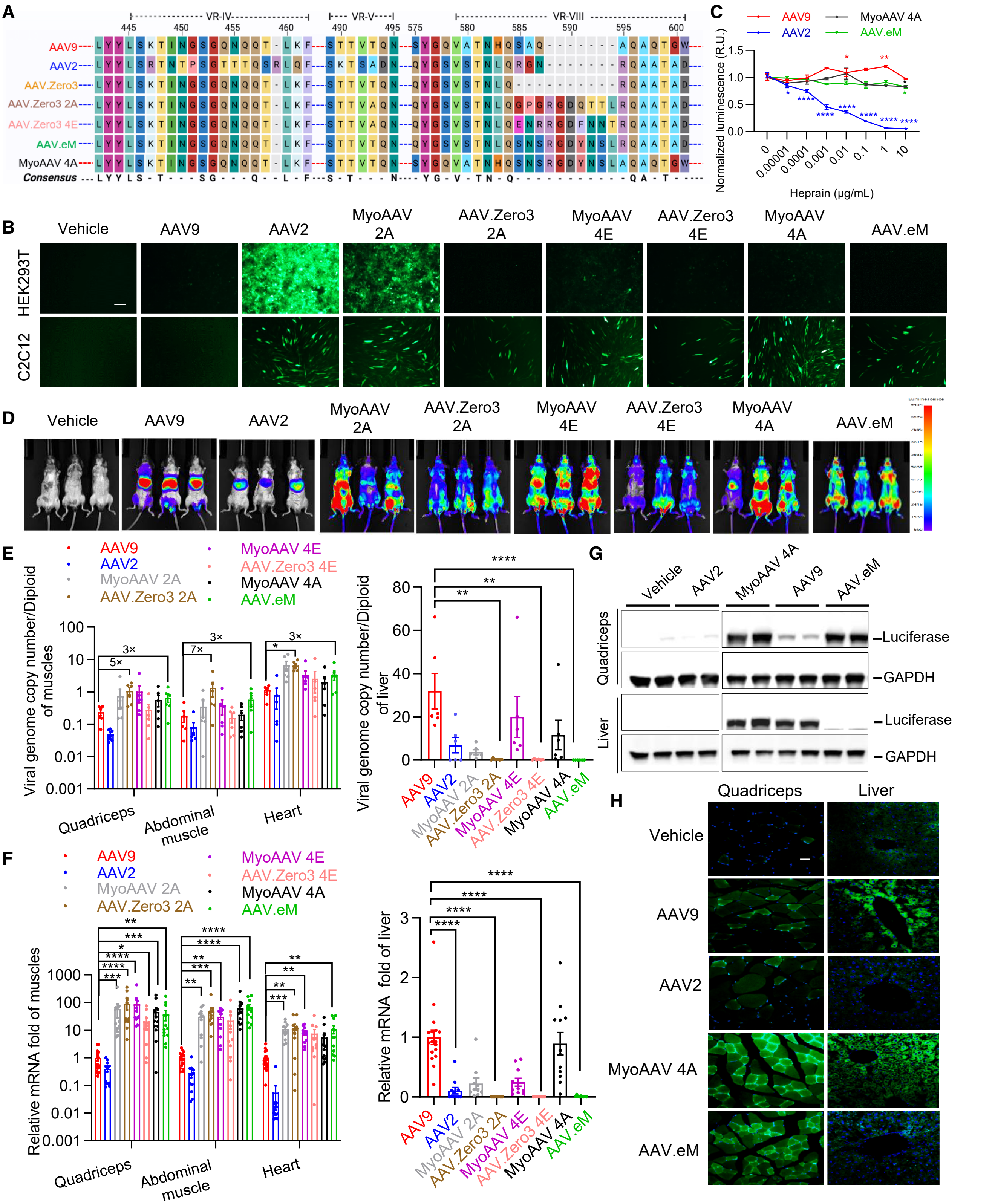


(legend on next page)

luciferase activity in the liver compared to AAV9 (Figures 1E, 1F, S1D, and S1E). Consistent with these observations, quantitative analysis showed that AAV.Zero1 and AAV.Zero3 yielded significantly fewer viral genome copies (Figure 1G) and lower levels of luciferase mRNA transcripts in the liver compared to AAV9 (Figures 1H and S1F). Western blotting analysis further confirmed that both chimeric variants produced markedly less luciferase protein in liver than AAV9 (Figures 1I and S1G). Beyond the liver, we also detected the biodistribution of all three novel capsids across multiple tissues, including quadriceps, abdominal muscle, heart, lung, spinal cord, brain, kidney, and eye. Both AAV.Zero1 and AAV.Zero3 demonstrated reduced viral genome copies and lower transgene expression in all organs examined at both low (Figures 1G–1I) and high (Figures S1F and S1G) viral doses. These findings indicate that AAV.Zero1 and AAV.Zero3 serve as promising platform capsids with broadly attenuated tissue tropism, providing an optimized foundation for further engineering of vectors with enhanced selectivity for target organs.

The capsid protein sequence of AAV.Zero1 differs from that of AAV2 only in the substituted VRs IV and V, which were replaced with the corresponding sequences from AAV9. This chimeric design resulted in a broad reduction of transduction efficiency across all organs examined compared to the parental AAV2 capsid. Remarkably, introduction of a single-point mutation (R585A) into AAV.Zero1—generating AAV.Zero2—restored viral transduction capacity and robust transgene mRNA expression in multiple muscular organs, including quadriceps, abdominal muscle, heart (Figures 1G–1I, S1F, and S1G), and eye (Figure S1F). These results indicate that in the context of an AAV2-based capsid already harboring AAV9-derived VRs IV and V, the residue R585 acts as a critical structural determinant that fine-tunes transduction efficiency across tissues, potentially by modulating receptor interactions that are distinct from the canonical HSPG-binding role described for R585 in wild-type AAV2. In contrast, AAV.Zero3, which lacks R585-N587 sequence, exhibited a low-transduction phenotype similar to AAV.Zero1 across all tissues, further supporting the essential role of this region as a molecular determinant of tropism.

### Enhanced muscle specificity achieved through the incorporation of RGD-containing peptides into a chimeric capsid

We next sought to engineer tissue-specific targeting properties into one of the low transduction capsids by the insertion of a peptide sequence. For enhanced muscle tropism, we integrated three previously characterized myotropic RGD (arginine-glycine-aspartic)-containing peptides,[34] 2A, 4E, and 4A, into AAV.Zero3 at site 585, generating new capsids AAV.Zero3 2A, AAV.Zero3 4E, and AAV.eM, respectively (Figure 2A). *In vitro* cell transduction assays demonstrated that all three modified vectors mediated detectable transduction of C2C12 myoblasts, albeit at lower efficiencies than AAV2 and reference MyoAAV serotypes. No transduction was observed in HEK293T, Huh-7, HelaRC32, and CHO-K1 cells (Figures 2B and S2A). Notably, quantitative viral genome analysis revealed a discrepancy between cellular entry and transgene expression, as vector genome copy number did not correlate with EGFP fluorescence intensity (cf. Figures 2B and S2B). Neutralization inhibition assays using anti-AAV2 and anti-AAV9 antibodies indicated that the peptide-modified variants retained serologic profiles similar to the parental AAV.Zero3, showing significant neutralization by anti-AAV2 antibodies and minimal inhibition by anti-AAV9 antibodies across all concentrations tested (Figures S2C and S2D). These results confirm that the introduced modifications did not alter the cross-reactive immune escape phenotype of the capsid, which remains antigenically closer to AAV2 than to AAV9. Interestingly, AAV.eM exhibited relatively more resistance to intravenous immunoglobulin (IVIg) compared to both AAV2 and AAV9 (Figure S2E). As pre-existing antibodies often target common receptor-binding epitopes, we first sought to determine if the critical heparan sulfate proteoglycan (HSPG) binding site of the parental AAV2 was fundamentally altered in AAV.eM, as this is a major target of human neutralizing antibodies. Further, we conducted heparin competition

**Figure 2. RGD-containing peptides enable chimeric capsid selectively target to muscle with high efficiency in C57BL/6J mice**

(A) Sequence alignments of VR-IV, VR-V and VR-VIII of the capsid variants containing RGD peptides 2A, 4E, and 4A inserted into AAV.Zero3 at R585. The blue dashed line represents the AAV2 backbone, while the red dashed line represents the AAV9 backbone. (B) Representative images of *in vitro* transduction in HEK293T and C2C12 cell lines were transduced at MOI = 1E5 with AAV9-, AAV2-, MyoAAV 2A-, AAV.Zero3 2A-, MyoAAV 4E-, AAV.Zero3 4E-, MyoAAV 4A-, or AAV.eM-CAG-Fluc-P2A-EGFP 72 h after transduction. Scale bars, 100 μm. (C) Heparin competition assay for AAV-mediated LacZ reporter transduction. Data are presented as luminescence normalized to vehicle-treated controls ($n = 3$ technical replicates). Statistical significance was determined by two-way ANOVA followed by comparisons of each heparin dilution group against the corresponding vehicle-treated control for each AAV capsid. All values represent means ± SEM. *$p < 0.05$, **$p < 0.01$, ****$p < 0.0001$. (D–F) Eight-week-old C57BL/6J mice were systemically injected with $2 \times 10^{11}$ vg per mouse AAV9-, AAV2-, MyoAAV 2A-, AAV.Zero3 2A-, MyoAAV 4E-, AAV.Zero3 4E-, MyoAAV 4A-, or AAV.eM-CAG-Fluc-P2A-EGFP. Representative whole body *in vivo* bioluminescence images 21 days post-injection. (D) The images for the Vehicle, AAV9, and AAV2 control groups are the same as those presented in Figure 1E. These images are derived from the same experimental batch and are reused here as a common internal control to enable a direct and consistent comparison with the novel capsids under investigation. Viral genome copy number per diploid from mice tissues, including quadriceps, abdominal muscle, and heart (left) and liver (right) detected by qPCR with standard curve. (E) For individual tissues, comparisons between AAV9 and each other capsid were performed using the Kruskal-Wallis test. ($n = 6$ biologically independent mice per group). **$p < 0.01$, ****$p < 0.0001$. Quantification of the *Fluc* mRNA in mice tissues, including quadriceps, abdominal muscle, and heart (left) and liver (right) at 21 days post-injection. (F) Comparisons between AAV9 ($n = 17$) and other capsids ($n = 11–12$) for each tissue were performed using the Kruskal-Wallis test. All samples ($n$ values) represent biologically independent mice. *$p < 0.05$, **$p < 0.01$, ***$p < 0.001$, ***$p < 0.001$. (G–H) Eight-week-old C57BL/6 J mice were systemically injected with 2E11 vg per mouse (∼8E12 vg/kg) AAV9-, AAV2-, MyoAAV 4A-, or AAV.eM-CAG-Fluc-P2A-EGFP. (G) Representative western blot images detecting luciferase and GAPDH of quadriceps and liver tissues 21 days post-injection. (H) Representative cross-section fluorescent images of quadriceps and liver tissues 21 days post-injection. Green: EGFP, blue: DAPI. Scale bars, 25 μm.

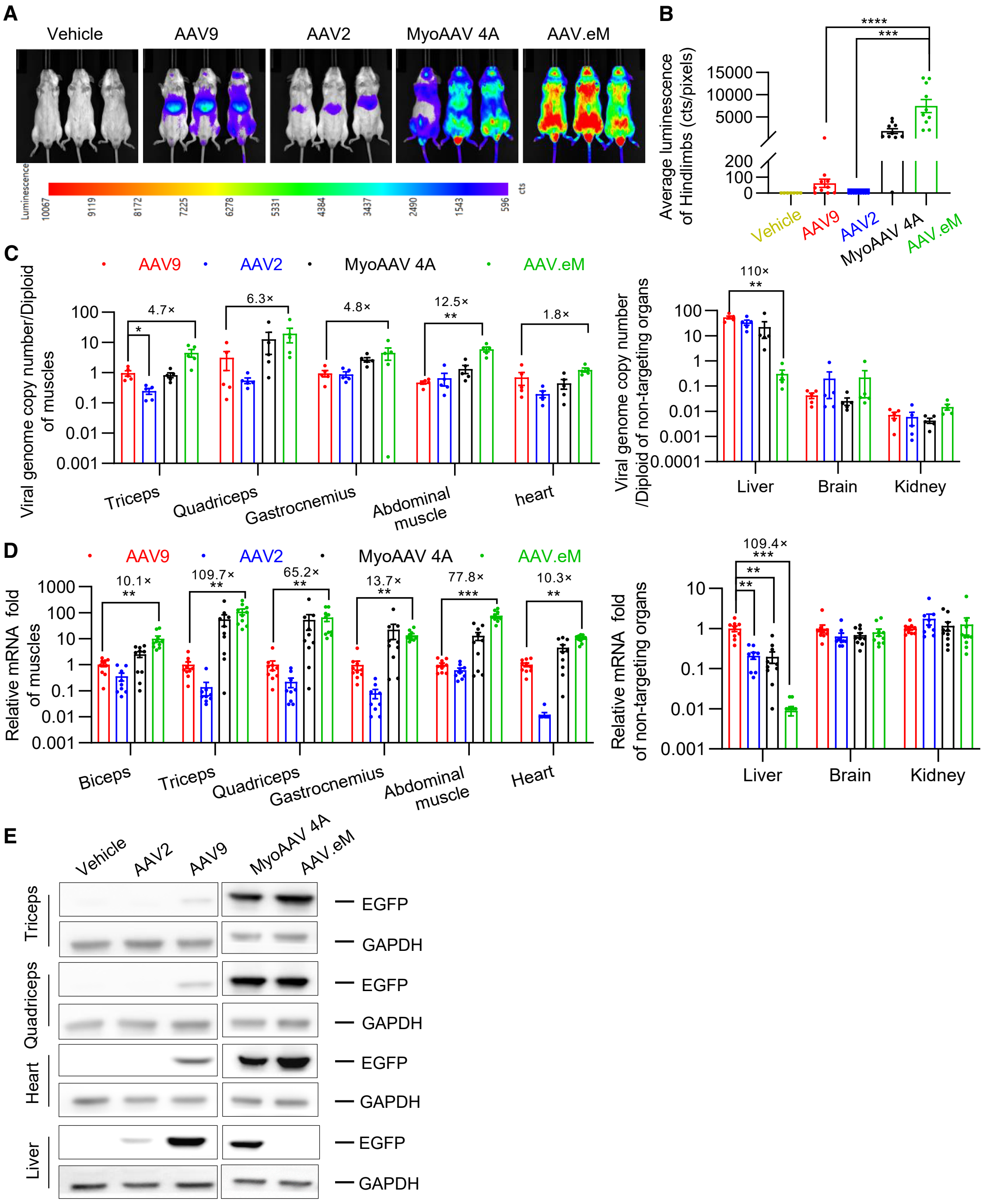


**Figure 3. AAV.eM is both efficient and specific in muscle-targeting across rodent species**

Eight-week-old BALB/c mice were systemically injected with $2 \times 10^{11}$ vg per mouse of AAV9-, AAV2-, MyoAAV 4A-, or AAV.eM-CAG-Fluc-P2A-EGFP and data were collected 21 days post-injection. (A) Representative whole body *in vivo* bioluminescence images. (B) Quantification of firefly luciferase luminescence from hindlimbs. The Kruskal-Wallis test was used for comparisons between AAV.eM and each other group. Sample sizes were $n = 10$ for each group, except for AAV2 ($n = 9$) and the control group ($n = 7$), ***$p < 0.001$, ****$p < 0.0001$. (C) Viral genome copy number per diploid from mouse tissues detected by qPCR using a standard curve. Comparisons between AAV9 and each other capsid across tissues were performed using ordinary one-way ANOVA, the Brown-Forsythe test and Welch's ANOVA, or the Kruskal-Wallis test, as appropriate for the data distribution and variance homogeneity ($n = 5$), *$p < 0.05$, **$p < 0.01$. (D) Quantification of the *Fluc* mRNA in biceps, triceps, quadriceps,

*(legend continued on next page)*

assay with AAV2, AAV9 MyoAAV 4A, and AAV.eM (Figure 2C). As expected, the infectivity of AAV2 decreased sharply with increasing heparin concentration. In contrast, AAV9, MyoAAV 4A, and AAV.eM showed no significant reduction in transduction across the same heparin gradient, maintaining nearly 100% activity. This result provides strong evidence that AAV.eM has undergone a qualitative change at the primary HSPG binding site compared to its AAV2 parent. This functional detachment from the canonical AAV2-HSPG interaction may provide a promising reason for why AAV.eM could evade a significant portion of pre-existing neutralizing antibodies prevalent in the human population that target this region.

Adult C57BL/6J mice were intravenously injected with $2 \times 10^{11}$ vg of AAV vectors, including parental capsid AAV9 or AAV2, novel chimeric capsid (AAV.Zero3 2A, AAV.Zero3 4E, and AAV.eM), or established myotropic capsid (MyoAAV 2A, MyoAAV 4E, and MyoAAV 4A), packaging with a CAG-luciferase-P2A-EGFP transgene cassette to investigate their transduction profiles and biodistributions *in vivo*. Three weeks post-injection, whole body and organ bioluminescence imaging shows that the novel RGD-modified AAV.Zero3 variants mediated luciferase expressions in muscle tissues comparable to that of the established MyoAAV counterparts, and significantly higher than that achieved with AAV2 or AAV9 (Figures 2D and S2F). Consistent with these observations, quantitative analyses demonstrated elevated viral genome copies (Figure 2E), luciferase mRNA (Figure 2F), and luciferase protein levels (Figures 2G and S2G) in muscle tissues from mice treated with both novel and established myotropic vectors. Fluorescence imaging of tissue sections further confirmed robust EGFP expression in quadriceps (Figure 2H). Notably, the novel AAV.Zero3-based vectors exhibited reduced luciferase activity in the liver compared to the MyoAAV controls, indicating preserved liver de-targeting (Figures 2E–2H and S2G). These results suggest that the insertion of RGD-containing peptides into the de-targeted AAV.Zero3 backbone enhances muscle-specific transduction while maintaining low hepatic tropism. This approach provides a promising strategy for developing synthetic capsids that exhibit high specificity for target tissues with concomitant reduction in off-target transduction.

### AAV.eM efficiently and specifically targets muscle across mouse species

A major challenge for engineering tissue-specific tropism of a vector is replicating transduction profiles across species. We chose AAV.eM as a candidate for further development and cross-species validation because it contains the 4A peptide, which has previously been shown to have cross-species myotropic properties in capsids.[25,34] After initial evaluation in C57BL/6J mice, we intravenously injected AAV9, AAV2, MyoAAV4A, or AAV.eM (packaged with a CAG-luciferase-P2A-EGFP cassette) to adult BALB/c mice at a dose of $2 \times 10^{11}$ vg per mouse ($\sim 8 \times 10^{12}$ vg/kg). Transduction efficiency and biodistribution were assessed 3 weeks post-injection (Figure 3A). Whole-organ bioluminescence imaging shows that AAV.eM mediated significantly higher luciferase activity in limb muscles compared to both parental AAV2 and AAV9, and the myotropic reference MyoAAV 4A (Figure 3B). Similarly, AAV.eM produced significantly higher viral genome copies per cell transduced (Figure 3C) and luciferase mRNA levels (Figure 3D) in skeletal muscles and heart compared to MyoAAV 4A. In non-target tissues, such as brain and kidney, all four vectors exhibited comparable, low-level transduction (Figures 3C and 3D). Furthermore, western blot result of EGFP confirmed stronger transgene expression by AAV.eM in triceps, quadriceps, and cardiac muscles, coupled with markedly reduced signal in the liver relative to other vectors (Figure 3E). Collectively, these findings suggest that AAV.eM shows proficient muscle targeting and minimal liver targeting in two distinct mouse strains. This supports the feasibility of engineering capsids with refined and translatable tissue tropism by incorporating functional peptides into a de-targeted parental backbone.

### AAV.eM shows high muscle transduction and low liver toxicity in NHPs

The translation of findings from rodent models to human applications remains a fundamental challenge in gene therapy, with NHPs serve as critical translational models for evaluating safety, biodistribution, and efficacy prior to clinical trials.[35,36] Given that capsids engineered for tissue-specific tropism in mice often fail to generalize to primates,[37–40] we examined the performance of our novel AAV.eM vector in cynomolgus macaques (*Macaca fascicularis*). Animals received i.v. injections of either MyoAAV 4A or AAV.eM, each carrying a CAG-luciferase-P2A-EGFP expression cassette, at a dosage of $3 \times 10^{13}$ vg/kg. Hepatotoxicity was monitored for 2 weeks post-injection by measuring serum levels of alanine aminotransferase (ALT) and aspartate aminotransferase (AST), two reliable biomarkers of liver injury. Administration of MyoAAV 4A resulted in ALT and AST levels peaking on day 3 at 17.9-fold and 13.2-fold above baseline, respectively (Figure 4A). In contrast, animals treated with AAV.eM kept ALT and AST levels comparable to pre-injection baselines throughout the observation period, suggesting a superior safety profile following systemic delivery. Neither vector induced significant changes in circulating creatine kinase (CK) levels, suggesting an absence of measurable muscle damage over the 14-day monitoring period (Figure 4A).

Following systemic administration, the single-stranded AAV DNA genome undergoes conversion into double stranded DNA to enable

gastrocnemius, abdominal muscle, and heart muscles as well as the liver, brain, and kidney. For comparisons between AAV9 and each other group within individual tissues, the appropriate statistical test (Brown-Forsythe/Welch's ANOVA or the Kruskal-Wallis test) was applied based on data distribution and variance homogeneity. Sample sizes were $n = 9$ for the AAV2 group and $n = 10$ for all other groups. $^{**}p < 0.01$, $^{***}p < 0.001$. (E) Representative western blot images detecting EGFP and GAPDH of triceps, quadriceps, heart, and liver. All samples ($n$ values) represent biologically independent mice.

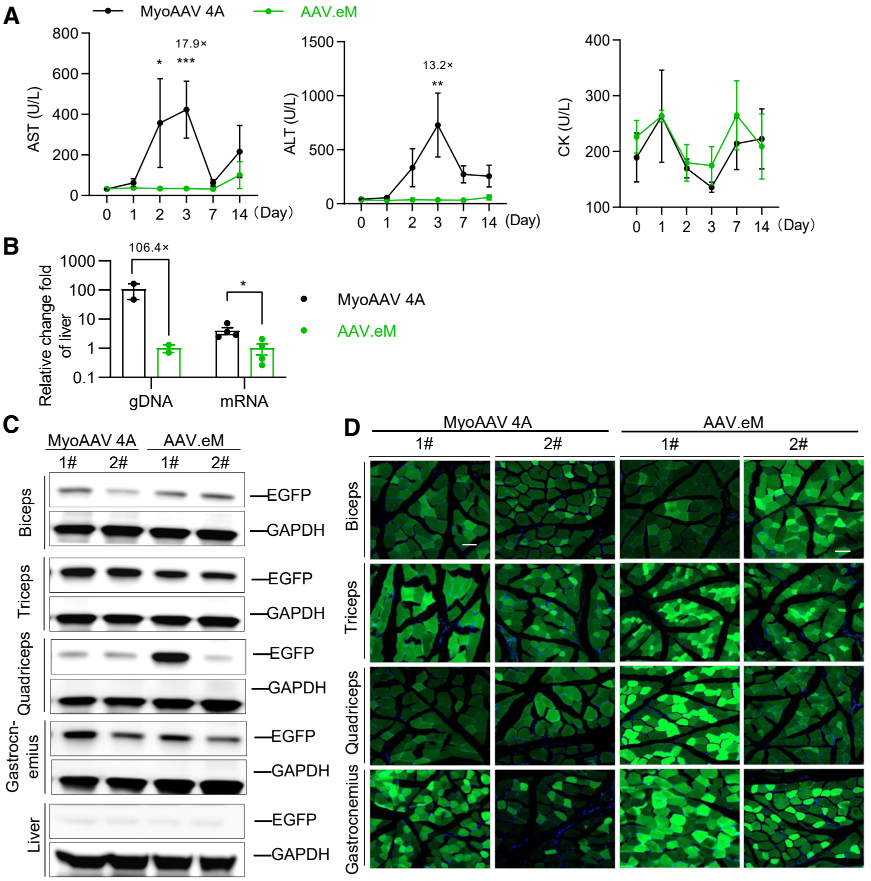


**Figure 4. AAV.eM causes less liver toxicity without sacrificing the transduction efficiency in non-human primates NHPs were given intravenous administration of 3 × 10$^{13}$ vg/kg of AAV.eM- or MyoAAV 4A-CAG-Fluc-P2A-EGFP**

(A) Hepatic enzyme (AST and ALT) and creatine kinase (CK) levels were monitored prior to and after dosing. The *p* values were calculated by Kruskal-Wallis test with Dunn's multiple comparisons test for each capsid. The number of monkeys were 5 for AST and ALT, and 3 for CK, $*p < 0.05$, $**p < 0.01$, $***p < 0.001$. AST, aspartate aminotransferase; ALT, alanine aminotransferase. (B) Quantification of relative vector genome per diploid genome and *Fluc* mRNA relative fold-change in the liver of NHPs at 28 days post-injection. The *p* value was calculated by unpaired Student's *t* test, the number of monkeys were 4 for mRNA and 2 for gDNA, $*p < 0.05$. (C) Representative western blot image detecting EGFP and GAPDH of biceps, triceps, quadriceps, gastrocnemius, and liver sampled at 14 days post-injection. (D) Representative cross-section fluorescent images of biceps, triceps, quadriceps, and gastrocnemius sampled 28 days post-injection. Green: EGFP, blue: DAPI, Scale bars, 100 μm. 1# and 2# represent two independent monkeys of each group, respectively.

transgene expression.[41] Analysis of liver tissue from injected macaques reveals that AAV.eM mediated significantly lower levels of viral genome accumulation and transgene expression compared to MyoAAV 4A (Figure 4B). In contrast, across multiple skeletal muscles, including biceps, triceps, quadriceps, and gastrocnemius, AAV.eM yielded vector genome copies and transgene expression comparable to those of MyoAAV 4A at 28 days post-injection (Figure S3A). In agreement with this, western blotting analysis confirmed robust EGFP protein expression in muscle tissues for both vectors, with minimal detection in the liver (Figure 4C). Transgene mRNA and protein levels correlated strongly with EGFP fluorescence imaging results in muscle sections (Figure 4D). To systematically evaluate biodistribution, we quantified viral genome copies across 17 tissues, encompassing muscular (e.g., biceps, triceps, quadriceps, gastrocnemius, tibialis anterior, soleus, abdominal wall, diaphragm, and heart) and non-muscular organs (liver, lung, brain, kidney, and spleen) (Figure S3B). AAV.eM showed comparable transduction as MyoAAV 4A in trunk muscles and cardiac tissues but notably less transduction in the liver (Figure S3B). AAV.eM demonstrated similar transduction efficiency to MyoAAV 4A in trunk and cardiac muscles but exhibited significantly reduced genome uptake in the liver. Gene expression profiling further indicated that AAV.eM and MyoAAV 4A produced similar transgene mRNA levels across most tissues. However, AAV.eM yielded approximately 116.0-fold and 90.1-fold lower expression in the liver and brain, respectively (Figure S3C). Together, these results demonstrate that AAV.eM maintains robust muscle tropism while conferring significantly reduced off-target transduction in the liver and brain, highlighting its improved specificity and safety profile in NHPs compared to the parent myotropic vector.

### A low-background capsid backbone is amenable to a variety of peptides directing tissue-specific transduction with minimal off-target effects

To assess whether the de-targeting capability of the AAV.Zero3 capsid is generalizable beyond MyoAAV-derived peptides, we engineered additional variants by incorporating distinct motifs: one derived from TGF-β (PG016)[42] and two selected from a randomized RGD peptide library (PG017 and PG018) identified from our previous screening (Figure S4A). All three novel capsids demonstrated muscle-targeting efficiency comparable to that of MyoAAV 4A in murine models while exhibiting markedly reduced transduction in the liver, brain, and kidney (Figures S4B–S4D). Consistent with these murine findings, systemic co-administration of these variants in NHPs confirmed markedly reduced liver tropism relative to conventional myotropic capsids (Tables S1–S6). Together, these results support the broad utility of the AAV.Zero3 scaffold for engineering peptide-directed tissue specificity with minimized background transduction across species. In addition to its favorable *in vivo* performance, AAV.eM also exhibits improved biophysical properties during vector production. Unlike MyoAAV 4A, which exhibits

concentration-dependent aggregation above $1 \times 10^{13}$ vg/mL as evidenced by DLS (dynamic light scattering) analysis. In contrast, AAV.eM retains a monodisperse profile even at ultra-high vector genome titers, highlighting its enhanced stability and reduced aggregation in our initial assessment under standard formulation conditions (Table S7).

### Systemic delivery of micro-dystrophin with AAV.eM produces functional correction in a DMD mouse model

To investigate the feasibility of using AAV.eM for *in vivo* delivery of therapeutic transgenes, we compared its delivery efficiency to AAV9, which is the delivery vector that has been used in clinical trials for DMD (NCT03362502 and NCT03368742).[43,44] We systemically administered AAV9 or AAV.eM containing a truncated function-complementing dystrophin (microdystrophin, μDys) transgene under the control of the muscle-specific *MHCK7* promoter to B10 DMD-KO mouse model (harboring a 4 bp deletion in *Dmd* exon 4).[45] At 14 weeks post-injection, the whole-limb grip strength assay indicates that the muscle strength of AAV.eM-μDys-injected mice improved compared to both DMD-KO vehicle- and AAV9-injected animals, indicating functional rescue of muscle function (Figure 5A). At 20 weeks post-injection, the serum CK level detected in AAV.eM-μDys-treated mice was significantly reduced compared to DMD-KO vehicle-treated group, indicating partial rescue of the severe muscle damage (Figure 5B). Biodistribution analysis revealed higher viral genome copies and μDys mRNA levels in multiple muscle tissues including biceps, triceps, quadriceps, gastrocnemius, abdominal muscle, and heart in AAV.eM-μDys-treated compared to the AAV9-μDys group, alongside reduced accumulation in off-target organs such as liver, lung, brain, and kidney (Figures 5C and 5D). Immunofluorescence staining confirmed robust, widespread dystrophin restoration in limb muscles of AAV.eM-μDys-injected mice, surpassing that achieved with AAV9-μDys (Figures 5E and 5F). These data establish that AAV.eM-μDys shows therapeutic efficacy in treating DMD-KO mice when delivered by systemic administration, which is superior to AAV9-μDys in terms of both targeting and off-target efficiency. This rationally engineered capsid produces enhanced muscle performance, reduced muscle damage, and elevated dystrophin mRNA and protein expression compared to the conventional AAV9 capsid, which are therapeutic targets that clinical trials are attempting to achieve in DMD patients. Thus, this vector and the design strategy used to produce it have the potential to deliver improved performance, reduced off-target transduction which can cause adverse events, and achieve therapeutic efficacy with lower dosages to improve the safety profile and reduce manufacturing costs.

## DISCUSSION

Recombinant AAV vectors have emerged as a leading platform for gene therapy across multiple organ systems.[46,47] The clinical potential of these vectors relies on achieving robust, tissue-specific transgene expression following systemic administration, while minimizing off-target effects and toxicity. Advances in AAV technology have resulted from synergistic improvements in both capsid engineering and vector genome design. The most common strategy to identify novel capsids with desired properties has been to screen a high-throughput library of capsids with random peptides inserted for directed evolution by a primary selection criterion (e.g., enhanced targeting to an organ), followed by more focused subsequent screenings of candidates to compare the properties of each AAV capsid directly in animals.[48–50] However, this approach is expensive and labor-intensive, and only produces incremental improvements in capsid performance with no guarantee that the capsid properties will translate across species. In contrast, rational design approaches have been undertaken based on the knowledge of capsid structures and mechanisms of AAV binding to cell surface receptors, internalization, and cellular trafficking. Rational design approaches primarily seek to alter capsid properties by engrafting designed peptides onto its surface.[51,52]

In this study, we employed rational design principles to engineer novel capsids, starting with two VRs swapping from AAV9 into AAV2 (creating AAV.Zero1) and alteration of the critical R585 residue (AAV.Zero2 containing an R585A substitution and AAV.Zero3 containing an R585GN deletion), followed by generating AAV.eM and other novel capsids via RGD-containing insertion at R585 into AAV.Zero3. AAV.Zero1 and AAV.Zero3 showed broadly lowered levels of transduction in major tissues compared to their parental capsids and specifically de-targeted the liver in two mouse strains, while AAV.Zero2 re-targets tissues with a single amino acid substitution. It is important to note that the pronounced effect of the R585 mutation observed here may be specific to the chimeric AAV.Zero background, in which the primary HSPG-binding residues (e.g., R484 and R487 in AAV2) have been ablated through VR swapping. This is consistent with previous findings that HSPG binding is a multi-residue determinant[53] Future work will focus on elucidating the precise molecular mechanisms underlying the de-targeting and retargeting profiles of our novel capsids. Investigation will be to determine how the VR swaps and the R585 mutations alter interactions with primary receptors (e.g., via using heparin-binding assays and competitive cell-binding studies) and with a broader repertoire to known or potential AAV co-receptors.

A key mechanistic insight from this study is provided by our heparin competition assays, which demonstrate that AAV.eM has lost the strong HSPG binding dependence characteristic of its AAV2 parent. This fundamental shift in primary receptor interaction offers a direct explanation for the observed de-targeting from tissues where AAV2 transduction is highly HSPG-dependent, such as the liver. Furthermore, AAV.eM also exhibited enhanced resistance to neutralization by human IVIg compared to both AAV2 and AAV9. This suggests that while AAV.eM retains antigenic similarity to AAV2 when tested against mono-specific antisera, its engineered surface effectively evades common human neutralizing antibodies—a finding of significant clinical relevance, as it may help overcome pre-existing humoral immunity in patients.

The reduced liver transduction of AAV.Zero3 and AAV.Zero1 compared to AAV2 implicates VRs IV and V in determining AAV2

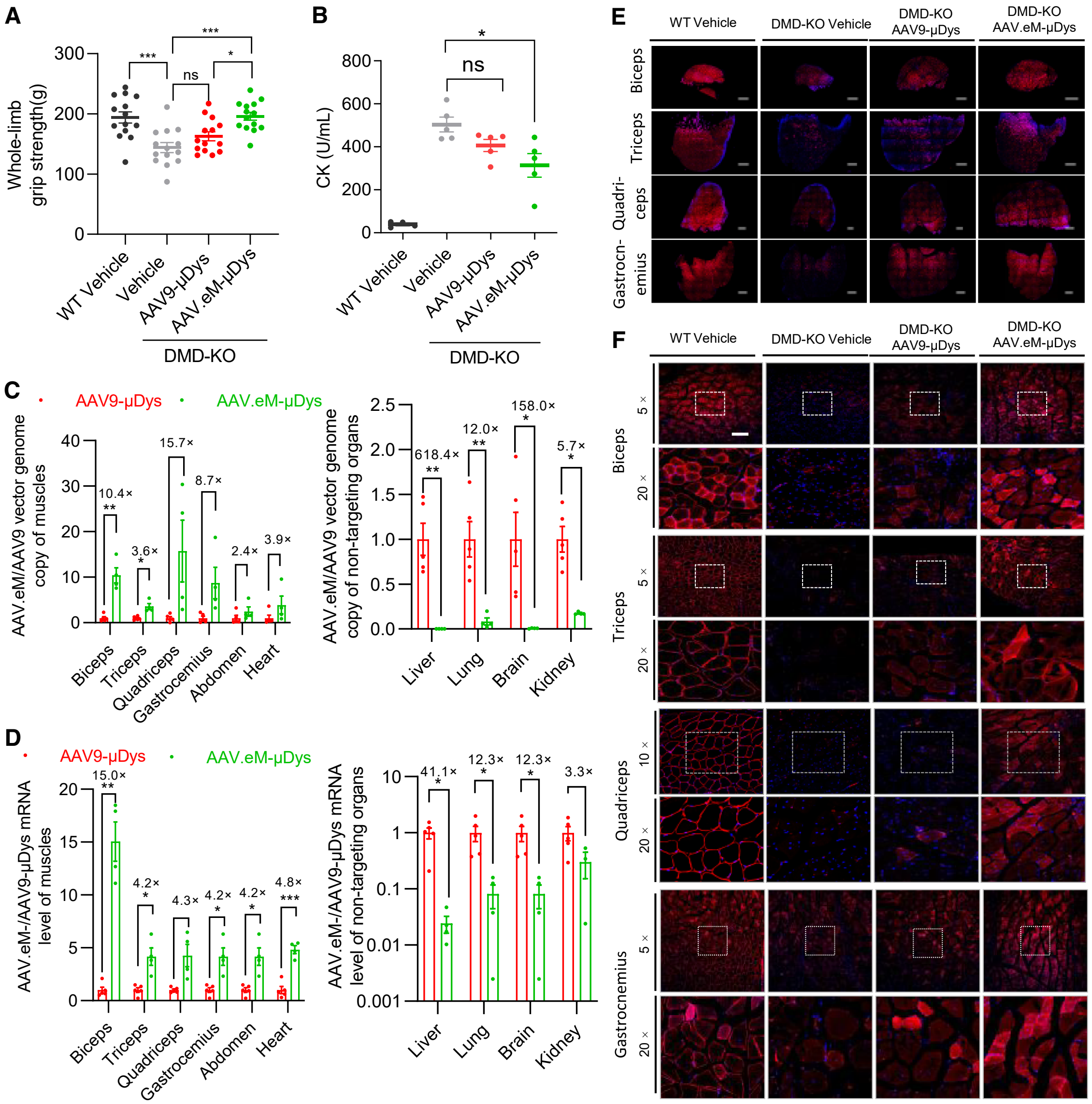


**Figure 5. Systemic injection of AAV.eM-MHCK7-microdystrophin results in widespread micro-dystrophin expression and effective restoration of muscle function in adult DMD mice 8-week-old wild-type B10 mice and B10 DMD-KO mice were systemically injected with vehicle or 2 $\times$ 10$^{13}$ vg/kg of either AAV9 or AAV.eM-MHCK7-microdystrophin (μDys)**

(A and B) The grip strength (A) and serum creatine kinase (CK) levels (B) were assessed 14 weeks post-injection. The $p$ values were calculated by one-way ANOVA with Tukey-Kramer multiple comparisons test, the number of mice were 14 of each group for grip strength, and 4–5 for CK concentration). *$p < 0.05$, ***$p < 0.001$, ns, not significant. (C–F) Quantification of relative vector genome copy number (C), mRNA expression (D), representative fluorescence images of cross-section whole mount (E) and zoom in (F) for μDys 20 weeks post-injection. The AAV.eM-μDys expression data are normalized to the relative expression levels of AAV9-μDys. Comparisons between the two groups for each tissue were performed using the Mann-Whitney $U$ test, Student's $t$ test, or Welch's $t$ test, as appropriate; the number of mice were 5 for AAV9-μDys, and 4 for AAV.eM-μDys except for kidney which is $n = 3$, *$p < 0.05$, **$p < 0.01$, ***$p < 0.001$. Red: μDys, blue: DAPI, Scale bars, 1 mm for (E), and 200 μm for (F).

liver tropism, consistent with studies highlighting analogous regions in other serotype (e.g., AAV.CAP-B10 in the context of AAV9).[54] Nonetheless, liver tropism is multifactorial, as evidenced by reports of other single-amino acid changes that modulate hepatic transduction across different capsids.[18,55–60] So far, no studies have been reported on optimizing off-target effects for tissues beyond the liver; therefore, our efforts in this area may provide valuable insights for the field.

The novel designed AAV.Zero3 2A, AAV.Zero3 4E, and AAV.eM capsids aimed to be muscle-tropic by inserting RGD-motif containing peptides at R585 in VR VIII of AAV.Zero3.[61,62] These showed

broadly similar profiles to the established myotropic vectors that the inserted peptides were identified from, so we examined the biodistribution of AAV.eM in NHPs because its inserted peptide has previously been shown to translate across species. We compared it to the established myotropic AAV vector MyoAAV 4A and found that the two capsids generally displayed comparable transduction efficiencies in various muscles of C57BL/6J mice, including limbs, abdominal muscle, and heart. This distribution pattern and high level of expression in muscle tissues, as measured by *in vivo* bioluminescence as well as DNA, mRNA, and protein levels, was replicated in the BALB/c mouse strain and NHPs, indicating robust and cross species-translatable muscle targeting properties. While AAV.eM showed promising biophysical properties in this study, future work will include a direct, controlled comparison using identical transgenes and a broader range of formulation buffers to conclusively establish its superior stability and manufacturability profile relative to other clinical-stage capsids. Importantly, the novel AAV.eM capsid showed a superior safety profile in NHPs compared to the established MyoAAV 4A capsid and functional correction delivering μDys in a mouse model of DMD. Thus, the AAV.eM capsid shows promise as a myotropic vector for systemic delivery of therapies to treat diseases that affect the musculature system or to achieve high serum levels of a protein using the muscles as an organ system for biomanufacturing. While this study demonstrates that AAV.eM mediates functional improvement superior to the clinical benchmark AAV9 in a DMD mouse model, a direct head-to-head functional comparison with other engineered myotropic capsids, such as MyoAAV 4A, in a therapeutic setting will be an important next step to fully delineate its comparative efficacy. Importantly, AAV.eM showed minimal liver transduction and did not induce increases in levels of circulating AST and ALT in NHPs, which are indicators of hepatotoxicity. In AAV gene therapy clinical trials, adverse events related to hepatotoxicity have been widely reported,[10,44] with increased incidents at higher vector doses. Thus, the AAV.eM and similarly engineered vectors can be employed to more efficiently deliver gene therapies to target tissues to achieve therapeutic efficacy without inducing liver damage. Additionally, the enhanced gene expression efficiency of this vector can allow for lower viral doses to be administered to achieve therapeutic effects, which is beneficial for both safety risks and manufacturing costs.

These novel capsids with broad low transduction efficiency levels for tissues allow for rational design principles such as peptide insertion and VR swapping to be applied in order to create novel capsids with specific properties such as enhanced transduction efficiency for specific tissues or cells and reduced off-target expression in unintended tissues. Many previous works have made a lot of effort in this filed but mainly focused on reducing liver de-targeting, so other peripheral tissues (e.g., brain and lung) remained unchanged or even had higher transgene expression levels compared with AAV9.[63,64] Here, AAV.eM inherited the systemic low-transduction in non-specific tissues by direct insertion of a well-studied muscle-targeting peptide into the backbone AAV.Zero3. We used this design principle to create three additional novel muscular-targeting capsids (PG016, PG017, and PG018) by insertion of RGD motif-containing peptides with sequence variety into the systemic de-targeting capsid backbone AAV.Zero3 to again produce muscle-specific tropism. These three capsid variants showed comparable muscle targeting as MyoAAV 4A and significantly lower transduction in the livers of mice and NHPs, suggesting that this engineering strategy to produce high tissue-specific expression with low off-target transduction that is conserved across species has broad applicability. Thus, this approach shows great potential to improve clinical therapeutic efficacy and safety profiles of rAAV capsids for gene therapies.

## MATERIALS AND METHODS

### Construction of the backbone capsid variants

The capsid sequences of AAV.Zero1, AAV.Zero2, and AAV.Zero3 were constructed by Gibson assembly (New England Biolabs [NEB], E5510S). The ∼5,000 bp plasmid backbone was recovered from restriction digestion of *SmiI* and *BshTI* of pAAV2-RC. Fragments A–F were amplified from the AAV2 Rep-Cap plasmid template with primers containing the replaced sequence of AAV9 (primer sequences are list in Table S8) (Takara Bio, R050Q). Primer pairs of each fragment are Cap-F/YJ69-R for A, YJ69-F/YJ72-R for B, YJ72-F/Cap-R for C, YJ72-F/247-R for D, 247-F/Cap-R for E, YJ72-F/248-R for F, and 248-F/Cap-R for G. The full sequence of AAV.Zero1 was assembled with fragments A, B, C, and plasmid backbone. The full sequence of AAV.Zero2 was assembled with fragments A, B, D, E, and plasmid backbone. The full sequence of AAV.Zero3 was assembled with fragments A, B, F, G, and plasmid backbone.

The peptide insertion variants were constructed in the same way as described above, with the differences being the PCR template (AAV.Zero3) and primers (see Table S8). Primer pairs of each fragment are Cap-F/249-R for H, 249-R/Cap-R for I, Cap-F/250-R for J, 250-R/Cap-R for K, and Cap-F/YJ107-R for L, and YJ107-R/Cap-R for M. The full sequence of AAV.Zero3 2A was assembled with fragments H, I, and plasmid backbone. The full sequence of AAV.Zero3 4E was assembled with fragments J, K, and plasmid backbone. The full sequence of AAV.Zero3 4A was assembled with fragments L, M, and plasmid backbone.

### Generation and screening of novel peptide-insertion capsid variants

To identify novel muscle-targeting capsid variants such as PG017 and PG018, we constructed and screened an AAV capsid library based on the AAV.Zero3 backbone, with random peptide insertions at VR-VIII. A GOI (gene of interest) plasmid library was first generated. The parental GOI plasmid contained inverted terminal repeat (ITR) sequences, a CAG promoter, an intron, and the AAV.Zero3 capsid sequence modified as follows: the fragment encoding amino acids T580 through the native stop codon was deleted, and the codon for T580 was silently mutated from ACC to ACT to introduce a novel BsrG I restriction site (TGTACA) operably linked to the downstream polyA sequence, facilitating subsequent cloning.

A pool of oligonucleotides encoding RGD-containing octapeptides was cloned into the capsid-encoding region at the site corresponding to amino acids 583–585 of the VP1 capsid protein (within VR-VIII). The native sequence at this locus (TNLQ583-R-Q585AATA) was replaced with RGD-containing octapeptides. All inserts were designed with an AG linker at the N terminus and either alanine (A) or arginine (R) at the C terminus, yielding the following 12 combinatorial patterns.

- TNLQ583-AGRGDXXXXXA/R-Q585AATA
- TNLQ583-AGXRGDXXXXA/R-Q585AATA
- TNLQ583-AGXXRGDXXXA/R-Q585AATA
- TNLQ583-AGXXXRGDXXA/R-Q585AATA
- TNLQ583-AGXXXXRGDXA/R-Q585AATA
- TNLQ583-AGXXXXXRGDA/R-Q585AATA

Long DNA primers encoding these peptide sequences were used to PCR-amplify fragments from the AAV.Zero3 template (Takara, R045Q). Purified nucleic acid fragments were ligated into BsrG I (Thermo Fisher Scientific, FD0933)-digested backbone vector by Gibson assembly (NEB, E2611L). Assembled products were treated with plasmid-safe DNase (Lucigen, E3101K) to remove residual linear fragments. The final GOI plasmid library was purified (US Everbright, UE-PCR-500) and verified by next-generation sequencing.

To ensure proper packaging of the library, we engineered a helper Rep-Cap plasmid containing stop codons introduced into the N-terminal regions of VP1, VP2, and VP3. This plasmid expresses functional Rep and AAP proteins but does not express VP1, VP2, or VP3 capsid proteins from the AAV.Zero3 backbone, ensuring that packaged capsids are derived exclusively from the GOI plasmid library. The Rep-Cap plasmid, GOI plasmid library, and pHelper plasmid were co-transfected into HEK 293T cells. Virus libraries were harvested 72 h post-transfection, purified by iodixanol density gradient ultracentrifugation, and titers were determined by qPCR (typical yields: $10^{12} - 10^{13}$ GC/mL). Purified virus libraries were administered systemically to NHPs for *in vivo* screening. Tissues were harvested 4 weeks (NHPs) post-injection, and mRNA was extracted and subjected to next-generation sequencing (Novogene) to identify variants enriched in muscle tissues. Candidate variants, including PG017 and PG018, were identified based on their high muscle enrichment ratios, subsequently cloned as individual capsids, and validated in follow-up *in vivo* studies.

#### Cell lines

The human embryonic kidney cell line HEK293T (research resource identifier [RRID]: CVCL_0063; source of the purchase: American type culture collection [ATCC], CRL-3216), human myoblast cell line C2C12 (RRID:CVCL_0188; source of the purchase: ATCC, CRL-1772), human cervical carcinoma cell line HelaRC32 (RRID: CVCL_B053; source of the purchase: ATCC, CRL-2972), and human hepatoma HuH-7 cell line (RRID:CVCL_0336; source of the purchase: National Collection of Authenticated Cell Cultures, SCSP-526) were cultured in Dulbecco's modified Eagle's medium (DMEM) (Thermo Fisher Scientific, C11995500BT) supplemented with 10% fetal bovine serum (FBS; Excell Bio, FSP500) and 1% penicillin-streptomycin (P/S; Biosharp, BL505A) and the hamster ovary cell line CHO-K1 (RRID:CVCL_0214; source of the purchase: Cobioer Gene Technology Co., CBP60296) was maintained in F12K media (Sigma-Aldrich, N3520-≈10X1L) supplemented with 10% FBS and 1% P/S. All cells were maintained at 37°C and 5% $CO_2$. All cells described earlier were tested negative for mycoplasma contamination.

#### Virus packaging

To package AAV viruses for cell transduction and animal injection, HEK293T cells were plated in 15 cm dishes at a density of $2 \times 10^7$ cells/dish. The next day, the rep-cap plasmid, pHelper plasmid, and ITR-containing plasmid AAV-CAG-Fluc-2A-eGFP were co-transfected into cells. At 72 h after transfection, cells were harvested by centrifugation at 500 × g for 10 min, resuspended in phosphate-buffered saline (PBS), and then the recombinant virus was released by freezing and thawing the cells three times. The crude lysate was clarified by centrifugation at 500 × g for 10 min and treated with benzonase at 250 U/mL final concentration at 37°C for 30 min. Virus was further purified by iodixanol step gradient and heparan sulfate affinity chromatography. AAV titers were quantified by qPCR with a standard curve or by TaqMan-based droplet digital PCR (ddPCR), targeting the ITR region. The viruses were stored at −80°C with titers ranging from 1E12 to 1E13 genome copies/mL.

#### AAV diameter

The particle size distribution of AAV preparations was assessed using DLS on the STUNNER nanoparticle analyzer (Unchained Labs, Pleasanton, CA, USA), following the manufacturer's standard protocol. Prior to analysis, AAV samples were diluted in 1 × PBS (pH 7.4) to an optimal concentration range (typically 1E12-1E13 vg/mL) to ensure accurate scattering signal and minimize multiple scattering effects. Measurements were conducted at 25°C using disposable cuvettes, with each sample analyzed in triplicate to assess reproducibility. The hydrodynamic diameter and polydispersity index (PDI) were derived from the autocorrelation function of the scattered light intensity and analyzed using cumulant fitting models. Results were reported as mean hydrodynamic diameter (*Z* average) and PDI, providing a quantitative evaluation of AAV particle size uniformity and aggregation status.

#### Neutralization inhibition assay

The *in vitro* rAAV reporter gene expression assay procedures are detailed as follows: seed HEK293T cells onto a 96-well plate at 3E4 cells/well in 100 μL media. Incubate the plate overnight at 37°C and 5% $CO_2$. To test the positive serum titer, dilute the test sera at ratios of 1:1, 1:5, 1: 10, 1:20, 1:40, 1: 80, 1: 160, 1:320, 1:640, 1: 1,280, 1:2,560, 1:5,120, 1: 10,240, and 1: 20,480 using DMEM. Mix 100 μL of each test serum dilution with wild-type AAV2 vectors or AAV9 vectors, separately, each at an MOI of 1E6. Incubate the mixtures for 1 h at 37°C to allow any neutralizing activity to occur.

Replace the old medium with 100 μL/well of the test serum-AAV vector mixture with 3 replicates for each mixture. Incubate the cell culture plate at 37°C for 48 h to allow AAV vector binding and entry into cells. Use a Fluc reporter assay to observe luciferase expression at 48 h post-infection. Calculate the anti-AAV-NAb titer by finding the serum dilution ratio that causes 50% inhibition of transduction efficacy. To test the neutralization resistance differences between various mutants, three or four dilutions were chosen for further experiments, which were no inhibition of transduction efficacy, half inhibition and lower limit of saturated inhibition. The specified operation is the same as described earlier.

For IVIg neutralization assay, it was conducted as previously reported (Wang et al.[65]). In brief, IVIg, 10% liquid Privigen, NDC 44206-437-91) was diluted 1:4 in DMEM, followed by 2-fold serial dilutions to 1:2,048. Each diluted IVIg sample was incubated with AAV2, AAV9, and AAV.eM, expressing lacZ in Huh7 cells at the MOI of 2,000, 10,000, and 10,000, respectively. Four wells were included for each dilution. Cells were then cultured in 5% FBS at 37°C in 5% $CO_2$ overnight. β-Galactosidase activity in cell lysate was measured using the Galacto-Star One-Step β galactosidase Reporter Gene Assay System (Thermo Fisher Scientific, cat. #T1014).

#### Heparin competition assay

HeLa cells were seeded into a 48-well plate (5E4 cells/well) and cultured overnight. HeLa cells were infected with Adv-AAV (MOI = 100 vg/cell) for 1 h. The AAVs.LacZ vectors were treated with different concentrations of Heparin for 30 min and were transduced to HeLa cells. The LacZ expression efficacy was quantified by luminescence values at 24 h post-transduction.

#### RNA isolation and RT-qPCR

Total RNA was extracted from samples using the TransZol Up Plus RNA kit (TransGen Biotech, ER501-01). The RNA concentration was determined using a Nanodrop (Thermo Fisher Scientific, 840–317400). Reverse transcription of 1 μg total RNA was performed with the EasyScript All-in-One First-Strand cDNA Synthesis SuperMix for qPCR (with one-step gDNA removal) kit (TransGen Biotech, AE341-03). Transgene expression was quantitated with 2 × SYBR Green qPCR master mix (Bimake, B21203) following the manufacturer's instructions. Procedures were run by StepOne Plus real-time PCR system (Applied Biosystems).

#### Quantification of barcodes by next-generation sequencing

RNA was reverse transcribed into cDNA using the HiScript III 1st-Strand cDNA Synthesis kit (with gDNA wiper) (Vazyme, R312-02). PCR was performed with barcoded primers and the Q5 Hot Start high-fidelity DNA polymerase (NEB, M0493L). PCR amplicons were purified by Hieff NGS DNA Selection Beads (Yeason, 12601ES08) and sequenced using Illumina NovaSeq X Plus (Novogene).

#### DNA isolation and quantification

Genomic DNA was extracted from tissue samples using the Hipure Universal DNA kit (Magen Biotech, D3018-03) in accordance with the manufacturer's protocol. Quantification of vector genome copies was carried out using ddPCR, targeting the firefly luciferase (*Fluc*) transgene and the endogenous *GAPDH* gene as a reference control gene (Figures 1G and S3B). ddPCR reactions were prepared using ddPCR Supermix for Probes (Bio-Rad, 1863024), with primers and a FAM (6-carboxyfluorescein)-labeled probe specific to the *Fluc* sequence or *GAPDH* (Table S8, synthesized by GENEWIZ). Droplet generation was performed using the Droplet Generator (Bio-Rad, QX200), followed by thermal cycling under standard conditions (95°C for 10 min, 40 cycles of 94°C for 30 s and 60°C for 1 min, and 98°C for 10 min). Droplets were then read on the QX200 Droplet Reader, and data were analyzed with QuantaSoft software to determine absolute copy numbers. Absolute number of *Fluc* transgene and *GAPDH* molecules in each sample was quantified using a standard curve generated by amplifying the linearized CAG-Fluc-P2A-EGFP plasmid or a *GAPDH* containing sequence in each run by qPCR (Figures 2E, 3D, and S3B).

#### Western blot

Protein lysates from tissue samples were prepared in radioimmunoprecipitation assay (RIPA) buffer (Beyotime Biotechnology, P0013B) and centrifuged to remove debris. Total protein concentrations were determined using the modified BCA (bicinchoninic acid) protocol (Sangon Biotech, C503051). Equal amounts of total protein from each sample were separated on 8%–16% SurePAGE precast polyacrylamide gels (GenScript, M00660). MOPS (3-(N-morpholino) propanesulfonic acid) running buffer (GenScript, M00680-500) was used for electrophoresis. All gels were transferred to polyvinylidene fluoride (PVDF) membranes in Western Rapid Transfer Buffer (Beyotime, P0572-2 L). Membranes were blocked for 1 h in 5% skim milk (Beyotime, P0216-300 g). All membranes were then incubated for 16 h at 4°C with primary antibody diluted in primary antibody dilution buffer (Beyotime, P0023A-100 mL). Primary antibodies used were against luciferase (1:2,000, Proteintech, 27986-1-AP) and GAPDH (1:2,000, Proteintech, 10494-1-AP). Blots were washed three times for 10 min each with Tris-buffered saline with Tween 20 (TBST) and then incubated with a goat anti-rabbit HRP-conjugated secondary antibody (Proteintech, SA00001-2) diluted 1:5,000 in blocking buffer for 2 h at room temperature (RT).

#### Animal experiments

All relevant procedures involving animals were reviewed and approved by the Institutional Animal Care and Use Committee (IACUC) of South China Normal University and the Institutional Animal Care and Use Committee of Guangzhou Huazhen Bioscience (permit no. HZ2019027). Male mice (6–8 weeks old) were obtained from Zhuhai BesTest Bio-Tech Co., Ltd., and housed under a 12-h light-dark cycle with *ad libitum* access to food and water. To evaluate the AAV transduction potential, mice were randomly assigned to experiments and transduced via i.v. injection (lateral tail vein) with a virus dose of 2E11 or 1E12 vg per mouse. Animals were euthanized 3 weeks after injection and tissues were harvested for the downstream transgene expression and biodistribution analyses. For *in vivo* screening, candidate capsids were evaluated in

separate experimental batches. To control the inter-batch variability, the internal control groups (AAV9, AAV2, and/or MyoAAV 4A) were included in every batch, and their data were pooled for analysis, whereas each test mutant was assessed in a single batch. For neutralization assays, rabbit serum containing polyclonal antibodies against AAV2 or AAV9 was used. Antibodies were generated by immunizing rabbit with corresponding empty capsids ($10^{11}$ vg/animal, subcutaneously) followed by three boosts at 2 weeks intervals. Serum was collected 2 weeks after the final boost. For NHP studies, 10 male, 4–5 years old, cynomolgus monkeys were purchased from Guangzhou Huazhen Biosciences Company Limited and housed in a controlled environment set to maintain a temperature of 18°C–26°C with a relative humidity of 60%–80%. AAVs were intravenously administered on day 0, and serum biochemistry was measured on days 0, 1, 2, 3, 7, 14, and 28. Muscle biopsies were obtained on day 14 and 28 under anesthesia using a muscle biopsy punch. Liver biopsies were obtained on day 28 under anesthesia using a liver biopsy needle with guidance from ultrasound.

### *In vivo* imaging

Mice were injected with 150 mg/kg of D-luciferin (Promega, E1605) and anesthetized with isoflurane before imaging. The bioluminescence images were acquired 10 min after D-luciferin injection with a rate of one image per 2 min using the AniView100 multi-modality animal *in vivo* imaging system (Biolight Biotechnology). Total and average radiance was measured from the same size of the region of interest (ROI) using GV 6000ProII software (Biolight Biotechnology). The highest captured radiance over imaging time was determined as the peak of the kinetic curve and picked for analysis.

### Serum biochemistry assays

Each blood collection (about 1.0 mL per time point) was performed from peripheral vein of each animal into low binding tube containing 10 μL 0.5 M (K2) EDTA and placed on wet ice until centrifugation. Samples were centrifuged (3,200 × g for 10 min at 2°C–8°C) within 1 h of collection. ALT, AST, and CK levels were measured using a clinical analyzer (Cobas e311, Roche).

### Immunofluorescence

Tissues harvested animals were fixed with 4% paraformaldehyde (PFA, Biosharp, XG1050) overnight at 4°C and washed 3 × 10 min with Dulbecco's PBS (DPBS). Fixed tissues were immersed in 30% sucrose at 4°C until submersion, embedded in OCT (optimum cutting temperature) compound (Tissue-Tek, Biosharp, BL557A), and frozen at −80°C. Tissues were sectioned using FS800A cryostat (RWD Minux) at a thickness of 10 μm. Sections were placed on poly-L-lysine-coated slide glasses, dried for at least 30 min, and then stored at −20°C until being analyzed. Frozen sections were air-dried for 10 min at 37°C prior to subsequent staining. For the microdystrophin immunostaining, tissue sections were incubated with a blocking solution consisting of PBS with 10% goat serum and 0.1% Triton X-100 for 30 min at RT. Next, the sections were incubated with a rabbit polyclonal primary antibody against human dystrophin (1:200 in PBS; Abcam, ab15277) for 2 h at RT. After washing the sections 3 × 5 min with PBS, the sections were incubated with Alexa Fluor 555-labeled Donkey Anti-Rabbit IgG (H + L) secondary antibody (1:1,000 in PBS; Beyotime, A0453) for an hour at RT. Nuclear counterstaining was performed with 4′,6-diamidino-2-phenylindole (DAPI). For the DAPI staining, tissue sections were washed for 3 × 5 min with PBST (PBS + 0.1% Tween 20) and then mounted with anti-fluorescence quenched sealing solution (DAPI included) (Beyotime, P0131). Signals were detected with a confocal microscope (Zeiss LSM 800).

### Statistical analysis and data visualization

Statistical analyses were carried out using GraphPad Prism v.9. For each dataset, normality was assessed using the Shapiro-Wilk test. For comparisons among multiple groups against a common control (e.g., AAV9), data passing the normality test were further analyzed using the Brown-Forsythe test to assess homogeneity of variances. Data with homogeneous variances were analyzed by ordinary one-way ANOVA followed by Tukey's post hoc test, while data with unequal variances were analyzed using Welch's ANOVA. Non-normally distributed data were analyzed using the Kruskal-Wallis test with Dunn's post hoc test. For comparisons between two groups, normally distributed data with equal variances were analyzed using Student's $t$ test, while those with unequal variances were analyzed using Welch's $t$ test. Non-normally distributed data were analyzed using the Mann-Whitney $U$ test. Data are presented as the mean ± standard error of the mean. A $p$ value of 0.05 or less was considered statistically significant in all experiments.

## DATA AND CODE AVAILABILITY

Source data for each relevant figure are provided in a source data file. The data that support the findings of this study are available from the corresponding author upon reasonable request.

## ACKNOWLEDGMENTS

This work was supported by grants from Shenzhen Medical Research Fund (D2301004).

## AUTHOR CONTRIBUTIONS

Conceptualization, H.L., Y.B., Y.Z., and Y.P.; investigation, Y.P., H.C., Y.Z., Z.D., J.C., K.T., X.C., D.Q., L.S., and X.T.; methodology, Y.P., H.C., Y.Z., Z.D., L.S., and J.C.; project administration, Y.B., Y.Z., and Y.P.; resources, H.L., Y.B., M.G., R.D., Z.Z., and Z.Y.; supervision, H.L., Y.B., and Y.P.; validation, Y.P. and Y.B.; visualization, Y.P. and H.C.; original draft writing, Y.P.; review and editing, G.G., H.L., Y.B., Y.P., Y.Z., Y.F., M.G., and R.D.

## DECLARATION OF INTERESTS

H.L., Y.P., Y.Z., H.C., Y.Z., Z.D., J.C., K.T., X.C., D.Q., L.S., X.T., Y.F., Y.B., Z.Z., and Z.Y. are employees of PackGene Biotech Inc. PackGene has filed patent applications related to the subject matter of this paper: WO2024138809A1, WO2024138811A1, PCT/CN2025/084668, and PCT/CN2025/085186. G.G. is a scientific cofounder of Voyager Therapeutics, Adrenas Therapeutics, and Aspa Therapeutics and holds equity in these companies.

## SUPPLEMENTAL INFORMATION

Supplemental information can be found online at https://doi.org/10.1016/j.omta.2026.201725.

## REFERENCES

1. Gao, G., Lu, Y., Calcedo, R., Grant, R.L., Bell, P., Wang, L., Figueredo, J., Lock, M., and Wilson, J.M. (2006). Biology of AAV serotype vectors in liver-directed gene

transfer to nonhuman primates. Mol. Ther. *13*, 77–87. https://doi.org/10.1016/j.ymthe.2005.08.017.

2. Wang, D., Tai, P.W.L., and Gao, G. (2019). Adeno-associated virus vector as a platform for gene therapy delivery. Nat. Rev. Drug Discov. *18*, 358–378. https://doi.org/10.1038/s41573-019-0012-9.
3. Zincarelli, C., Soltys, S., Rengo, G., and Rabinowitz, J.E. (2008). Analysis of AAV serotypes 1-9 mediated gene expression and tropism in mice after systemic injection. Mol. Ther. *16*, 1073–1080. https://doi.org/10.1038/mt.2008.76.
4. Wang, J.-H., Gessler, D.J., Zhan, W., Gallagher, T.L., and Gao, G. (2024). Adeno-associated virus as a delivery vector for gene therapy of human diseases. Signal Transduct. Targeted Ther. *9*, 78. https://doi.org/10.1038/s41392-024-01780-w.
5. Mimuro, J., Mizukami, H., Hishikawa, S., Ikemoto, T., Ishiwata, A., Sakata, A., Ohmori, T., Madoiwa, S., Ono, F., Ozawa, K., and Sakata, Y. (2013). Minimizing the inhibitory effect of neutralizing antibody for efficient gene expression in the liver with adeno-associated virus 8 vectors. Mol. Ther. *21*, 318–323. https://doi.org/10.1038/mt.2012.258.
6. Kelsic, E.D., and Church, G.M. (2019). Challenges and opportunities of machine-guided capsid engineering for gene therapy. Cell Gene Ther. Insights *5*, 523–536.
7. Morales, L., Gambhir, Y., Bennett, J., and Stedman, H.H. (2020). Broader Implications of Progressive Liver Dysfunction and Lethal Sepsis in Two Boys following Systemic High-Dose AAV. Mol. Ther. *28*, 1753–1755. https://doi.org/10.1016/j.ymthe.2020.07.009.
8. (2020). High-dose AAV gene therapy deaths. Nat. Biotechnol. *38*, 910. https://doi.org/10.1038/s41587-020-0642-9.
9. Lek, A., Wong, B., Keeler, A., Blackwood, M., Ma, K., Huang, S., Sylvia, K., Batista, A.R., Artinian, R., Kokoski, D., et al. (2023). Death after High-Dose rAAV9 Gene Therapy in a Patient with Duchenne's Muscular Dystrophy. N. Engl. J. Med. Overseas. Ed. *389*, 1203–1210. https://doi.org/10.1056/NEJMoa2307798.
10. Muscular Dystrophy (2025). Sarepta reports death of DMD patient given gene therapy Elevidys. https://musculardystrophynews.com/news/sarepta-reports-death-dmd-patient-elevidys-gene-therapy/?preview_id=96612.
11. Duan, D. (2023). Lethal immunotoxicity in high-dose systemic AAV therapy. Mol. Ther. *31*, 3123–3126. https://doi.org/10.1016/j.ymthe.2023.10.015.
12. Jun, X., and Phillip, W.L.T. (2025). AAV-RNAi constructs promote DRG toxicity. Mol. Ther. *33*, 21–22.
13. Hordeaux, J., Buza, E.L., Dyer, C., Goode, T., Mitchell, T.W., Richman, L., Denton, N., Hinderer, C., Katz, N., Schmid, R., et al. (2020). Adeno-Associated Virus-Induced Dorsal Root Ganglion Pathology. Hum. Gene Ther. *31*, 808–818. https://doi.org/10.1089/hum.2020.167.
14. Daniel Stone, M.A., and Jerome, K.R. (2023). Breaching the blood-brain barrier: AAV triggers dose-dependent toxicity in the brain. Mol. Ther., Methods Clin. Dev. *31*, 101105. https://doi.org/10.1016/j.omtm.2023.09.001.
15. Guo, Y., Chen, J., Ji, W., Xu, L., Xie, Y., He, S., Lai, C., Hou, K., Li, Z., Chen, G., and Wu, Z. (2023). High-titer AAV disrupts cerebrovascular integrity and induces lymphocyte infiltration in adult mouse brain. Mol. Ther., Methods Clin. Dev. *31*, 101102. https://doi.org/10.1016/j.omtm.2023.08.021.
16. Duan, D. (2018). Systemic AAV micro-dystrophin gene therapy for Duchenne muscular dystrophy. Mol. Ther. *26*, 2337–2356.
17. Büning, H., Huber, A., Zhang, L., Meumann, N., and Hacker, U. (2015). Engineering the AAV capsid to optimize vector–host-interactions. Curr. Opin. Pharmacol. *24*, 94–104.
18. Goertsen, D., Flytzanis, N.C., Goeden, N., Chuapoco, M.R., Cummins, A., Chen, Y., Fan, Y., Zhang, Q., Sharma, J., Duan, Y., et al. (2022). AAV capsid variants with brain-wide transgene expression and decreased liver targeting after intravenous delivery in mouse and marmoset. Nat. Neurosci. *25*, 106–115.
19. Qian, R., Xiao, B., Li, J., and Xiao, X. (2021). Directed evolution of AAV serotype 5 for increased hepatocyte transduction and retained low humoral seroreactivity. Mol. Ther., Methods Clin. Dev. *20*, 122–132.
20. Arjomandnejad, M., Dasgupta, I., Flotte, T.R., and Keeler, A.M. (2023). Immunogenicity of Recombinant Adeno-Associated Virus (AAV) Vectors for Gene Transfer. BioDrugs *37*, 311–329. https://doi.org/10.1007/s40259-023-00585-7.
21. Agbandje-McKenna, M., and Kleinschmidt, J. (2011). AAV capsid structure and cell interactions. Methods Mol. Biol. *807*, 47–92. https://doi.org/10.1007/978-1-61779-370-7_3.
22. Nonnenmacher, M., Wang, W., Child, M.A., Ren, X.Q., Huang, C., Ren, A.Z., Tocci, J., Chen, Q., Bittner, K., Tyson, K., et al. (2021). Rapid evolution of blood-brain-barrier-penetrating AAV capsids by RNA-driven biopanning. Mol. Ther., Methods Clin. Dev. *20*, 366–378. https://doi.org/10.1016/j.omtm.2020.12.006.
23. Vogt, N. (2021). DELIVERing transgenes into muscle. Nat. Methods *18*, 1275.
24. Chen, X., Ravindra Kumar, S., Adams, C.D., Yang, D., Wang, T., Wolfe, D.A., Arokiaraj, C.M., Ngo, V., Campos, L.J., Griffiths, J.A., et al. (2022). Engineered AAVs for non-invasive gene delivery to rodent and non-human primate nervous systems. Neuron *110*, 2242–2257.e6. https://doi.org/10.1016/j.neuron.2022.05.003.
25. Stanton, A.C., Lagerborg, K.A., Tellez, L., Krunnfusz, A., King, E.M., Ye, S., Solomon, I.H., Tabebordbar, M., and Sabeti, P.C. (2023). Systemic administration of novel engineered AAV capsids facilitates enhanced transgene expression in the macaque CNS. Méd. Sur *4*, 31–50.e8. https://doi.org/10.1016/j.medj.2022.11.002.
26. Ojala, D.S., Sun, S., Santiago-Ortiz, J.L., Shapiro, M.G., Romero, P.A., and Schaffer, D.V. (2018). In Vivo Selection of a Computationally Designed SCHEMA AAV Library Yields a Novel Variant for Infection of Adult Neural Stem Cells in the SVZ. Mol. Ther. *26*, 304–319. https://doi.org/10.1016/j.ymthe.2017.09.006.
27. Liguore, W.A., Domire, J.S., Button, D., Wang, Y., Dufour, B.D., Srinivasan, S., and McBride, J.L. (2019). AAV-PHP.B Administration Results in a Differential Pattern of CNS Biodistribution in Non-human Primates Compared with Mice. Mol. Ther. *27*, 2018–2037. https://doi.org/10.1016/j.ymthe.2019.07.017.
28. Gonzalez, T.J., Simon, K.E., Blondel, L.O., Fanous, M.M., Roger, A.L., Maysonet, M.S., Devlin, G.W., Smith, T.J., Oh, D.K., Havlik, L.P., et al. (2022). Cross-species evolution of a highly potent AAV variant for therapeutic gene transfer and genome editing. Nat. Commun. *13*, 5947. https://doi.org/10.1038/s41467-022-33745-4.
29. Shen, X., Storm, T., and Kay, M.A. (2007). Characterization of the Relationship of AAV Capsid Domain Swapping to Liver Transduction Efficiency. Mol. Ther. *15*, 1955–1962. https://doi.org/10.1038/sj.mt.6300293.
30. Adachi, K., Enoki, T., Kawano, Y., Veraz, M., and Nakai, H. (2014). Drawing a high-resolution functional map of adeno-associated virus capsid by massively parallel sequencing. Nat. Commun. *5*, 3075. https://doi.org/10.1038/ncomms4075.
31. Opie, S.R., Warrington, K.H., Agbandje-McKenna, M., Zolotukhin, S., and Muzyczka, N. (2003). Identification of Amino Acid Residues in the Capsid Proteins of Adeno-Associated Virus Type 2 That Contribute to Heparan Sulfate Proteoglycan Binding. J. Virol. *77*, 6995–7006. https://doi.org/10.1128/jvi.77.12.6995-7006.2003.
32. Earley, J., Piletska, E., Ronzitti, G., and Piletsky, S. (2023). Evading and overcoming AAV neutralization in gene therapy. Trends Biotechnol. *41*, 836–845. https://doi.org/10.1016/j.tibtech.2022.11.006.
33. Schulz, M., Levy, D.I., Petropoulos, C.J., Bashirians, G., Winburn, I., Mahn, M., Somanathan, S., Cheng, S.H., and Byrne, B.J. (2023). Binding and neutralizing anti-AAV antibodies Detection and implications for rAAV-mediated gene therapy. Mol. Ther. *31*, 616–630.
34. Tabebordbar, M., Lagerborg, K., Stanton, A., King, E., Ye, S., Tellez, L., Krunnfusz, A., Tavakoli, S., Widrick, J., Messemer, K., et al. (2021). Cell Directed evolution of a family of AAV capsid variants enabling potent muscle-directed gene delivery. Cell *16*, 4919–4938.e22. https://doi.org/10.1016/j.cell.2021.08.028.
35. Bohlen, M.O., McCown, T.J., Powell, S.K., El-Nahal, H.G., Daw, T., Basso, M.A., Sommer, M.A., and Samulski, R.J. (2020). Adeno-Associated Virus Capsid-Promoter Interactions in the Brain Translate from Rat to the Nonhuman Primate. Hum. Gene Ther. *31*, 1155–1168. https://doi.org/10.1089/hum.2020.196.
36. Katz, M.G., Fargnoli, A.S., Weber, T., Hajjar, R.J., and Bridges, C.R. (2017). Use of adeno-associated virus vector for cardiac gene delivery in large-animal surgical models of heart failure. Hum. Gene Ther. Clin. Dev. *28*, 157–164.
37. Hordeaux, J.,W.Q., Katz, N., Buza, E.L., Bell, P., and Wilson, J.M. (2018). The Neurotropic Properties of AAV-PHP.B Are Limited to C57BL/6J Mice. Mol. Ther. *26*, 664–668. https://doi.org/10.1016/j.ymthe.2018.01.018.
38. Huang, Q., Chan, K.Y., Tobey, I.G., Chan, Y.A., Poterba, T., Boutros, C.L., Balazs, A.B., Daneman, R., Bloom, J.M., Seed, C., and Deverman, B.E. (2019). Delivering genes across the blood-brain barrier: LY6A, a novel cellular receptor for

AAV-PHP.B capsids. PLoS One *14*, e0225206. https://doi.org/10.1371/journal.pone.0225206.

39. Bunuales, M., Garduno, A., Chillon, M., Bosch, A., Gonzalez-Aparicio, M., Espelosin, M., Garcia-Gomara, M., Rico, A.J., Garcia-Osta, A., Cuadrado-Tejedor, M., et al. (2024). Characterization of brain transduction capability of a BBB-penetrant AAV vector in mice, rats and macaques reveals differences in expression profiles. Gene Ther. *31*, 455–466. https://doi.org/10.1038/s41434-024-00466-w.

40. Batista, A.R., King, O.D., Reardon, C.P., Davis, C., Gray-Edwards, H., Philip, V., Gray-Edwards, H., Aronin, N., Lutz, C., Landers, J., and Sena-Esteves, M. (2020). Ly6a Differential Expression in Blood–Brain Barrier Is Responsible for Strain Specific Central Nervous System Transduction Profile of AAV-PHP.B. Hum. Gene Ther. *31*, 90–102.

41. Berry, G.E., and Asokan, A. (2016). Cellular transduction mechanisms of adeno-associated viral vectors. Curr. Opin. Virol. *21*, 54–60. https://doi.org/10.1016/j.coviro.2016.08.001.

42. Campbell, M.G., Cormier, A., Ito, S., Seed, R.I., Bondesson, A.J., Lou, J., Marks, J.D., Baron, J.L., Cheng, Y., and Nishimura, S.L. (2020). Cryo-EM Reveals Integrin-Mediated TGF-β Activation without Release from Latent TGF-β. Cell *180*, 490–501.e16.

43. Inc., S.B. (2017). Microdystrophin Gene Transfer Study in Adolescents and Children With DMD. https://ucla.clinicaltrials.researcherprofiles.org/trial/NCT03368742.

44. National Library of Medicine US (2017). A Study to Evaluate the Safety and Tolerability of PF-06939926 Gene Therapy in Duchenne Muscular Dystrophy (ClinicalTrials.gov). https://clinicaltrials.gov/study/NCT03362502.

45. Zheng, J., Lou, J., Li, Y., Qian, P., He, W., Hao, Y., Xue, T., and Song, Y.-H. (2022). Satellite cell-specific deletion of Cipc alleviates myopathy in mdx mice. Cell Rep. *39*, 110939. https://doi.org/10.1016/j.celrep.2022.110939.

46. Valdmanis, P.N., and Kay, M.A. (2017). Future of rAAV gene therapy: platform for RNAi, gene editing, and beyond. Hum. Gene Ther. *28*, 361–372.

47. Rodríguez-Márquez, E., Meumann, N., and Büning, H. (2021). Adeno-associated virus (AAV) capsid engineering in liver-directed gene therapy. Expet Opin. Biol. Ther. *21*, 749–766.

48. Kondratov, O., Kondratova, L., Mandel, R.J., Coleman, K., Savage, M.A., Gray-Edwards, H.L., Ness, T.J., Rodriguez-Lebron, E., Bell, R.D., Rabinowitz, J., et al. (2021). A comprehensive study of a 29-capsid AAV library in a non-human primate central nervous system. Mol. Ther. *29*, 2806–2820.

49. Davidsson, M., Wang, G., Aldrin-Kirk, P., Cardoso, T., Nolbrant, S., Hartnor, M., Mudannayake, J., Parmar, M., and Björklund, T. (2019). A systematic capsid evolution approach performed in vivo for the design of AAV vectors with tailored properties and tropism. Proc. Natl. Acad. Sci. USA *116*, 27053–27062.

50. Yang, L., Jiang, J., Drouin, L.M., Agbandje-Mckenna, M., Chen, C., Qiao, C., Pu, D., Hu, X., Wang, D.Z., Li, J., and Xiao, X. (2009). A myocardium tropic adeno-associated virus (AAV) evolved by DNA shuffling and in vivo selection. Proc. Natl. Acad. Sci. USA *106*, 3946–3951.

51. Chen, Y.H., Chang, M., and Davidson, B.L. (2009). Molecular signatures of disease brain endothelia provide new sites for CNS-directed enzyme therapy. Nat. Med. *15*, 1215–1218.

52. Asokan, A., Conway, J.C., Phillips, J.L., Li, C., Hegge, J., Sinnott, R., Yadav, S., DiPrimio, N., Nam, H.J., Agbandje, M., et al. (2010). Reengineering a receptor footprint of adeno-associated virus enables selective and systemic gene transfer to muscle. Nat. Biotechnol. *28*, 79–82.

53. Perabo, L., Goldnau, D., White, K., Endell, J., Boucas, J., Humme, S., Work, L.M., Janicki, H., Hallek, M., Baker, A.H., and Büning, H. (2006). Heparan sulfate proteoglycan binding properties of adeno-associated virus retargeting mutants and consequences for their in vivo tropism. J. Virol. *80*, 7265–7269. https://doi.org/10.1128/jvi.00076-06.

54. Zolotukhin, S., Trivedi, P.D., Corti, M., and Byrne, B.J. (2021). Scratching the surface of RGD-directed AAV capsid engineering. Mol. Ther. *29*, 3099–3100.

55. Kern, A., Schmidt, K., Müller, O.J., Müller, O.J., Wobus, C.E., Bettinger, K., Von der Lieth, C.W., King, J.A., and Kleinschmidt, J.A. (2003). Identification of a Heparin-Binding Motif on Adeno-Associated Virus Type 2 Capsids. J. Virol. *77*, 11072–11081. https://doi.org/10.1128/jvi.77.20.11072-11081.2003.

56. Zinn, E., Unzu, C., Schmit, P.F., Turunen, H.T., Zabaleta, N., Sanmiguel, J., Fieldsend, A., Bhatt, U., Diop, C., Merkel, E., et al. (2022). Ancestral library identifies conserved reprogrammable liver motif on AAV capsid. Cell Rep. Med. *3*, 100803. https://doi.org/10.1016/j.xcrm.2022.100803.

57. Cabanes-Creus, M., Navarro, R.G., Liao, S.H.Y., Scott, S., Carlessi, R., Roca-Pinilla, R., Knight, M., Baltazar, G., Zhu, E., Jones, M., et al. (2023). Characterization of the humanized FRG mouse model and development of an AAV-LK03 variant with improved liver lobular biodistribution. Mol. Ther., Methods Clin. Dev. *28*, 220–237. https://doi.org/10.1016/j.omtm.2022.12.014.

58. Wang, D., Li, S., Gessler, D.J., Xie, J., Zhong, L., Li, J., Tran, K., Van Vliet, K., Ren, L., Su, Q., et al. (2018). A Rationally Engineered Capsid Variant of AAV9 for Systemic CNS-Directed and Peripheral Tissue-Detargeted Gene Delivery in Neonates. Mol. Ther., Methods Clin. Dev. *9*, 234–246. https://doi.org/10.1016/j.omtm.2018.03.004.

59. Xing, R., Xu, M., Reil, D., Destefano, A., Cui, M., Liu, N., Liang, J., Xu, G., Luo, L., Xu, M., et al. (2025). A single amino acid variant in the variable region I of AAV capsid confers liver detargeting. PloS Pathogens *21*, e1013533. https://doi.org/10.1371/journal.ppat.1013533.

60. Elad Firnberg, S.K.S.T., Yost, S.A., Giles, A.R., Choi, H., Foltz, S.J., Qian, R., Smith, J.B., Heithoff, B., Elliott, K., Hou, C.-F.D., et al. (2025). Directed evolution of liver-detargeted AAV vectors for systemic gene delivery to skeletal muscle and heart. Mol. Ther., Methods Clin. Dev. *33*, 101571. https://doi.org/10.1016/j.omtm.2025.101571.

61. George, L.A., Ragni, M.V., Rasko, J.E.J., Raffini, L.J., Samelson-Jones, B.J., Ozelo, M., Hazbon, M., Runowski, A.R., Wellman, J.A., Wachtel, K., et al. (2020). Long-term follow-up of the first in human intravascular delivery of AAV for gene transfer: AAV2-hFIX16 for severe hemophilia B. Mol. Ther. *28*, 2073–2082.

62. Wang, D., Li, S., Gessler, D.J., Xie, J., Zhong, L., Li, J., Tran, K., Van Vliet, K., Ren, L., Su, Q., et al. (2018). A rationally engineered capsid variant of AAV9 for systemic CNS-directed and peripheral tissue-detargeted gene delivery in neonates. Mol. Ther., Methods Clin. Dev. *9*, 234–246.

63. El Andari, J., Renaud-Gabardos, E., Tulalamba, W., Weinmann, J., Mangin, L., Pham, Q.H., Hille, S., Bennett, A., Attebi, E., Bourges, E., et al. (2022). Semirational bioengineering of AAV vectors with increased potency and specificity for systemic gene therapy of muscle disorders. Sci. Adv. *23*, 38. https://doi.org/10.1126/sciadv.abn4704.

64. Vu Hong, A., Suel, L., Petat, E., Dubois, A., Le Brun, P.R., Guerchet, N., Veron, P., Poupiot, J., and Richard, I. (2024). An engineered AAV targeting integrin alpha V beta 6 presents improved myotropism across species. Nat. Commun. *15*, 7965. https://doi.org/10.1038/s41467-024-52002-4.

65. Wang, D., Zhong, L., Li, M., Li, J., Tran, K., Ren, L., He, R., Xie, J., Moser, R.P., Fraser, C., et al. (2018). Adeno-Associated Virus Neutralizing Antibodies in Large Animals and Their Impact on Brain Intraparenchymal Gene Transfer. Mol Ther Methods Clin Dev *11*, 65–72. https://doi.org/10.1016/j.omtm.2018.09.003.

OMTA, Volume *34*

## Supplemental information

# Engineering novel AAV capsids by broadly attenuated and subsequent muscle-specific tropism in mice and NHPs

**Yue Pan, Yujian Zhong, Huan Chen, Youwei Zhang, Zhiyong Dai, Junlin Chen, Keqin Tan, Xiaoqu Chen, Danlan Qiu, Longxiang Sheng, Xinping Tan, Ying Fan, Ye Bu, Zexin Zhou, Zhiming Yang, Rui Duan, Min Guan, Guangping Gao, and Huapeng Li**

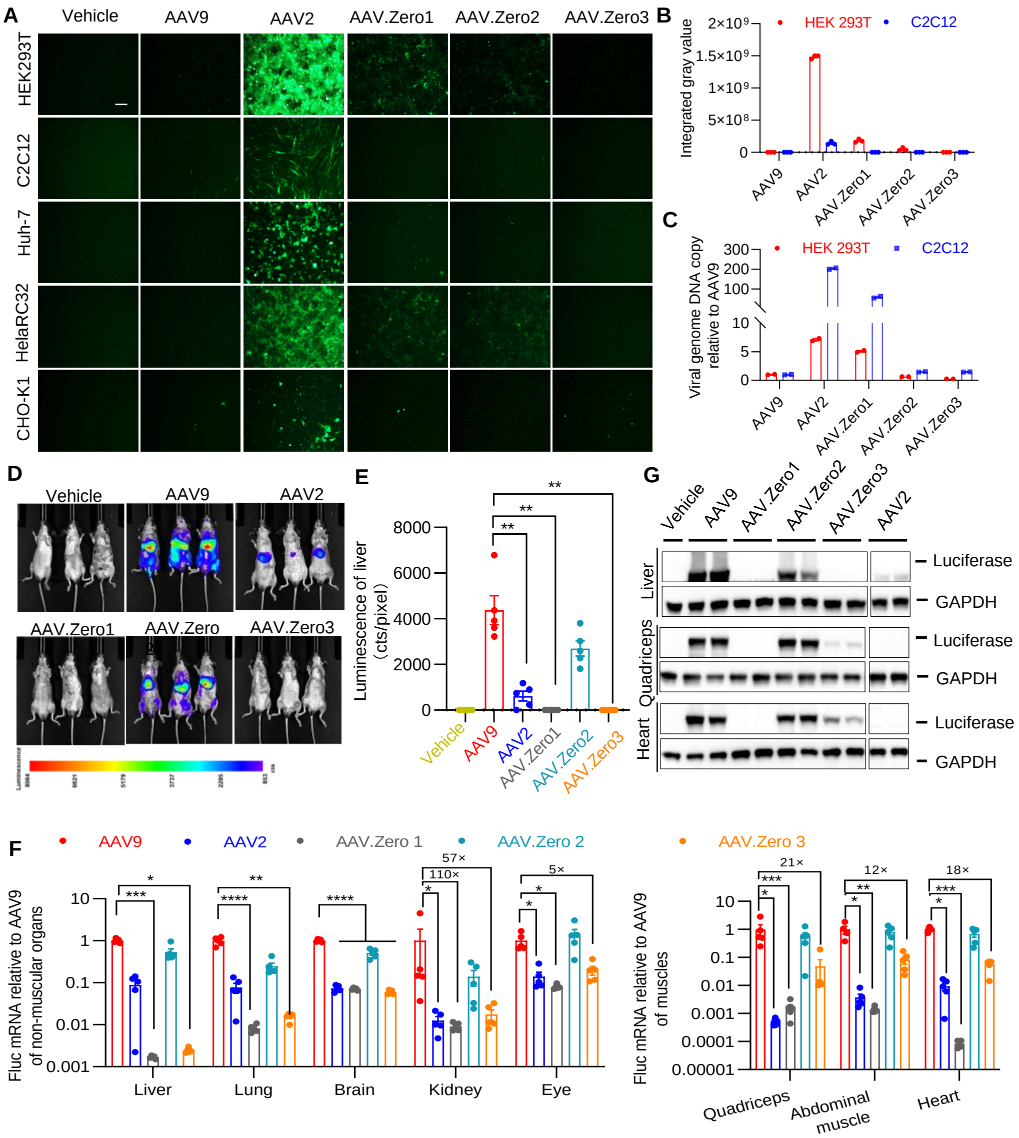
A
Vehicle
AAV9
AAV2
AAV.Zero1
AAV.Zero2
AAV.Zero3
HEK293T
C2C12
Huh-7
HelaRC32
CHO-K1
B
Integrated gray value
HEK 293T
C2C12
C
Viral genome DNA copy relative to AAV9
D
E
Luminescence of liver (cts/pixel)
G
Liver
Quadriceps
Heart
Luciferase
GAPDH
F
AAV.Zero 1
AAV.Zero 2
AAV.Zero 3
Fluc mRNA relative to AAV9 of non-muscular organs
Liver
Lung
Brain
Kidney
Eye
Fluc mRNA relative to AAV9 of muscles
Quadriceps
Abdominal muscle
Heart
57×
110×
5×
21×
12×
18×

**Figure S1. The tropisms of chimeric capsid variants systematically decreased even at higher virus dose**

A–C. HEK293T, C2C12, Huh-7, HelaRC32, and CHO-K1 cell lines being transduced with AAV9-, AAV2-, AAV.Zero1-, AAV.Zero2-, or AAV.Zero3-CAG-Fluc-P2A-EGFP at MOI=1E5 after 120 hours. A. Representative images of each treatment. Scale bar: 100 μm. B. Quantification of the integrated gray value of the EGFP fluorescence strength in HEK 293T and C2C12 cells (n=3). C. Quantification of relative virus genome copy numbers in HEK 293T and C2C12 cells (n=2). D–G. 8-week-old C57BL/6J mice were systemically injected with $1 \times 10^{12}$ vg per mouse (~ $4 \times 10^{13}$ vg/kg) of AAV9-, AAV2-, AAV.Zero1-, AAV.Zero2-, or AAV.Zero3-CAG-Fluc-P2A-EGFP and data were collected 21 days post-injection. Representative whole body *in vivo* bioluminescence images (D). Quantification of firefly luciferase luminescence from liver (E) and quantification of fold-difference in *Fluc* mRNA expression in various tissues compared with normalized AAV9 (F). Comparisons between AAV9 and each of the other groups were performed using the Brown-Forsythe test and Welch's ANOVA, or the Kruskal-Wallis test, as appropriate for the data distribution and variance. (n=5 biologically independent animals, except for AAV9 abdominal muscle n=4), *$p < 0.05$, **$p < 0.01$, ***$p < 0.001$ and ****$p < 0.0001$. Representative western blot images detecting luciferase and GAPDH in quadriceps, heart, and liver (G).

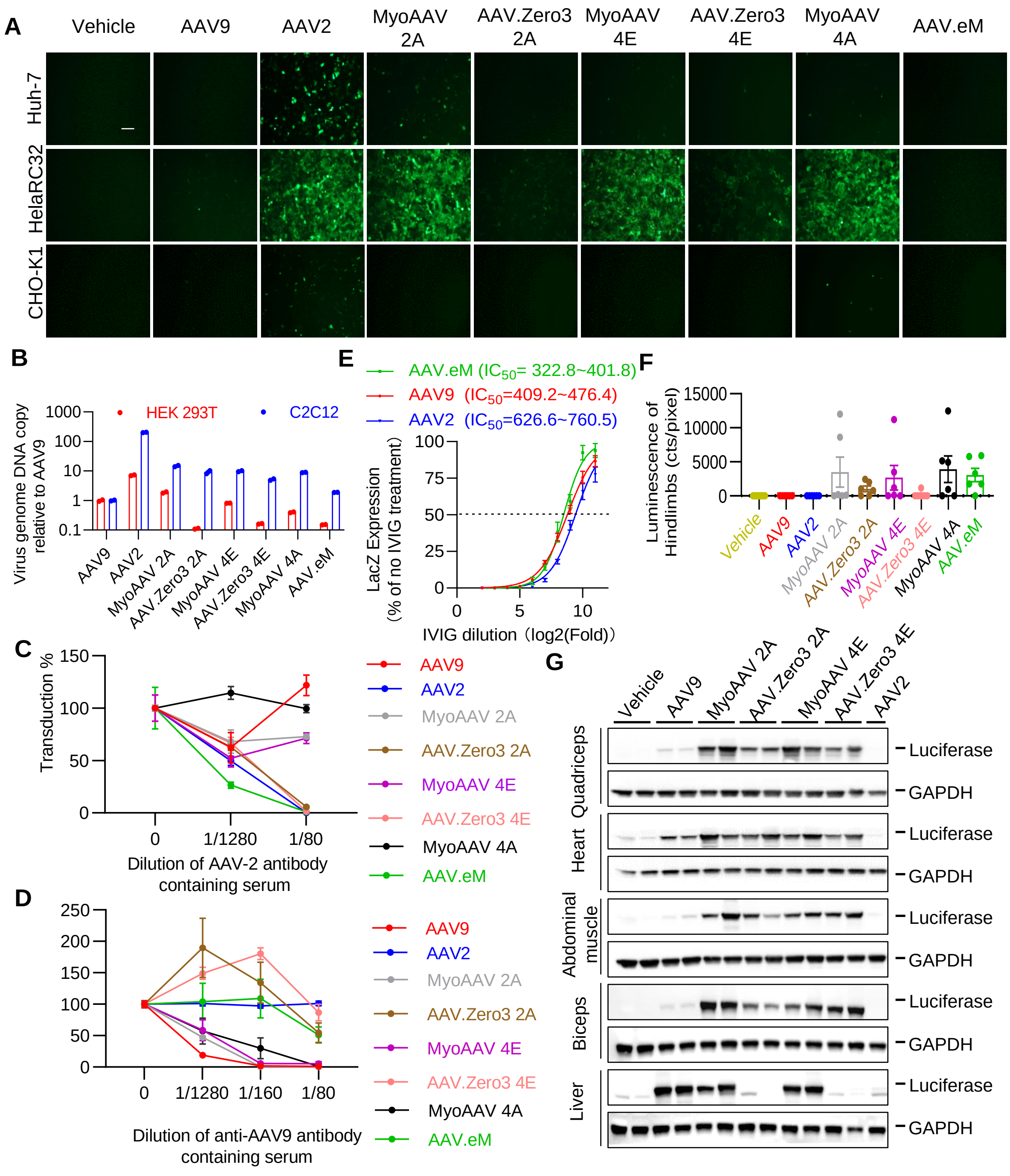

A
Vehicle
AAV9
AAV2
MyoAAV 2A
AAV.Zero3 2A
MyoAAV 4E
AAV.Zero3 4E
MyoAAV 4A
AAV.eM
Huh-7
HelaRC32
CHO-K1
B
Virus genome DNA copy relative to AAV9
HEK 293T
C2C12
1000
100
10
1
0.1
AAV9
AAV2
MyoAAV 2A
AAV.Zero3 2A
MyoAAV 4E
AAV.Zero3 4E
MyoAAV 4A
AAV.eM
E
AAV.eM ($IC_{50}$= 322.8~401.8)
AAV9 ($IC_{50}$=409.2~476.4)
AAV2 ($IC_{50}$=626.6~760.5)
LacZ Expression (% of no IVIG treatment)
IVIG dilution (log2(Fold))
F
Luminescence of Hindlimbs (cts/pixel)
15000
10000
5000
0
Vehicle
AAV9
AAV2
MyoAAV 2A
AAV.Zero3 2A
MyoAAV 4E
AAV.Zero3 4E
MyoAAV 4A
AAV.eM
C
Transduction %
0
1/1280
1/80
Dilution of AAV-2 antibody containing serum
AAV9
AAV2
MyoAAV 2A
AAV.Zero3 2A
MyoAAV 4E
AAV.Zero3 4E
MyoAAV 4A
AAV.eM
D
0
1/1280
1/160
1/80
Dilution of anti-AAV9 antibody containing serum
G
Vehicle
AAV9
MyoAAV 2A
AAV.Zero3 2A
MyoAAV 4E
AAV.Zero3 4E
AAV2
Quadriceps
Heart
Abdominal muscle
Biceps
Liver
Luciferase
GAPDH

**Figure S2. RGD-containing peptides enable chimeric capsid selectively target to muscle with high efficiency in C57BL/6J mice**

A. Representative images Huh-7, HelaRC32, and CHO-K1 cell lines 72 hours after being transfected with AAV9-, AAV2-, MyoAAV 2A-, AAV.Zero3 2A-, MyoAAV 4E-, AAV.Zero3 4E-, MyoAAV 4A-, or AAV.eM-CAG-Fluc-P2A-EGFP at MOI=1E5. Scale bar: 100 μm. B. Quantification of relative virus genome copy numbers in HEK 293T and C2C12 cells transduced with AAV9-, AAV2-, MyoAAV 2A-, AAV.Zero3 2A-, MyoAAV 4E-, AAV.Zero3 4E-, MyoAAV 4A-, or AAV.eM-CAG-Fluc-P2A-EGFP at MOI=1E5 (n=2). C. Different dilutions of AAV2-antibody containing serum were incubated with constant amounts of virus at MOI=1E6 and tested for neutralization of transduction using an *in vitro* assay (n=4 for left panel and n=6 for right panel). D. Different dilutions of AAV9-antibody containing serum were incubated with constant amounts of virus at MOI=1E6 and tested for neutralization of transduction using an *in vitro* assay (n=3–6). E. Neutralization assay of AAV2, AAV9, MyoAAV 4A and AAV.eM using IVIG (n=4 wells), the confidence interval of $IC_{50}$ is 95%. F-G. 8-week-old C57BL/6J mice were systemically injected with $2 \times 10^{11}$ vg per mouse of AAV9-, AAV2-, MyoAAV 2A-, AAV.Zero3 2A-, MyoAAV 4E-, AAV.Zero3 4E-, MyoAAV 4A-, or AAV.eM-CAG-Fluc-P2A-EGFP- and data were collected 21 days post-injection. Quantification of the firefly luciferase luminescence intensity from hindlimbs (n=6 biologically independent animals) (F). Representative western blot images detecting luciferase and GAPDH in quadriceps, heart, abdominal muscle, biceps, and liver (G).

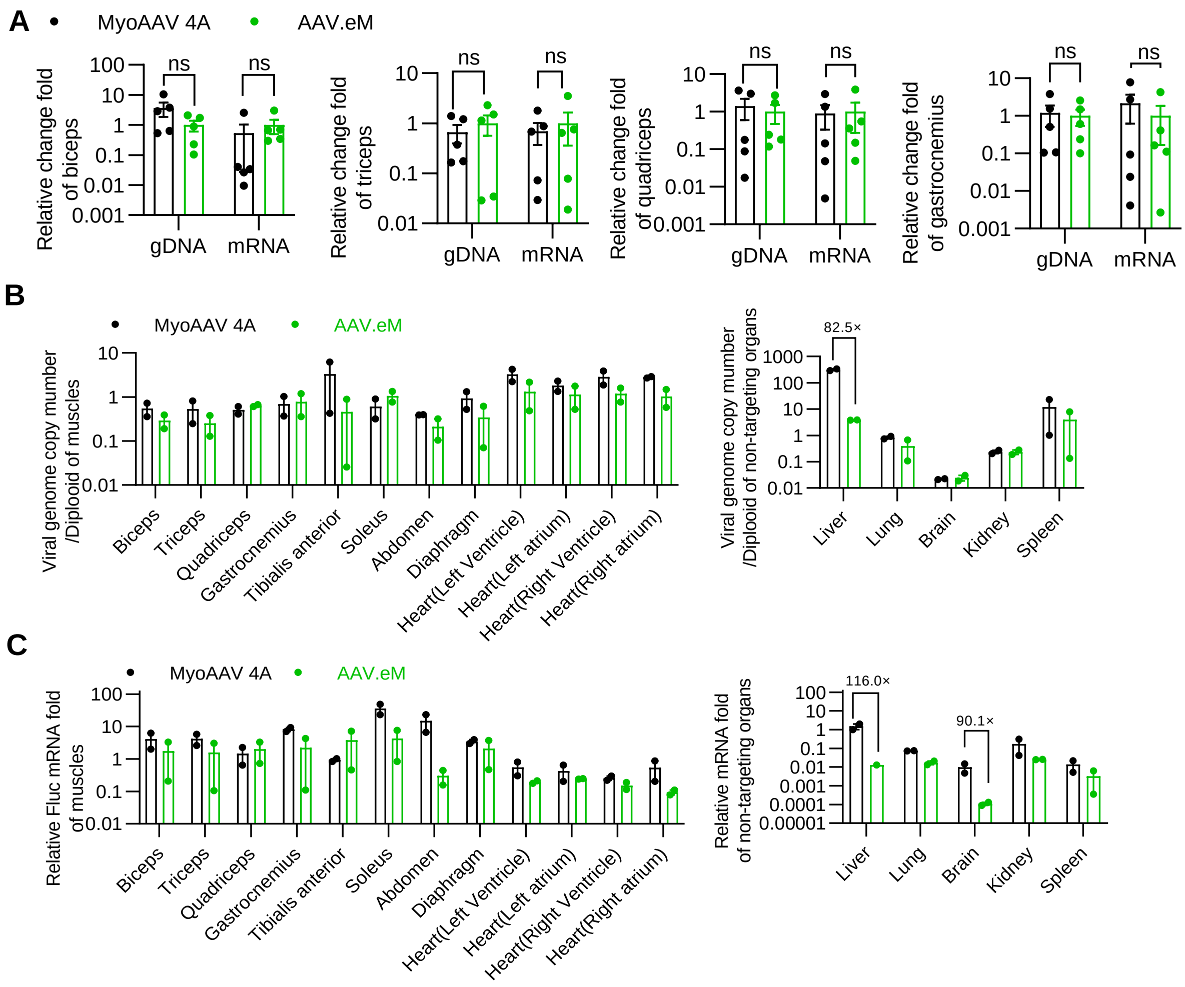

A
MyoAAV 4A
AAV.eM
Relative change fold of biceps
Relative change fold of triceps
Relative change fold of quadriceps
Relative change fold of gastrocnemius
ns
gDNA
mRNA
B
Viral genome copy number /Diploid of muscles
Viral genome copy number /Diploid of non-targeting organs
82.5×
Biceps
Triceps
Quadriceps
Gastrocnemius
Tibialis anterior
Soleus
Abdomen
Diaphragm
Heart(Left Ventricle)
Heart(Left atrium)
Heart(Right Ventricle)
Heart(Right atrium)
Liver
Lung
Brain
Kidney
Spleen
C
Relative Fluc mRNA fold of muscles
Relative mRNA fold of non-targeting organs
116.0×
90.1×

**Figure S3. AAV.eM exhibits less off-target transduction and comparable muscle-specific targeting compared with MyoAAV 4A**

A–C. NHPs were given intravenous administration of $3 \times 10^{13}$ vg/kg of MyoAAV 4A- or AAV.eM-CAG-Fluc-P2A-EGFP. Relative change fold of viral gDNA copy and *Fluc* mRNA in biceps, triceps, quadriceps and gastrocnemius sampled 28 days after virus injection (A). Comparisons between two groups were performed using Student's *t*-test, Welch's *t*-test, or the Mann-Whitney U test, as appropriate for the data distribution and variance. (n=5 biologically independent animals), ns: not significant. The biceps, triceps, quadriceps, gastrocnemius, tibialis anterior, soleus, abdominal muscle, diaphragm, heart, liver, lung, brain, kidney, and spleen were sampled 28 days after virus injection (n=2 biologically independent animals). Absolute quantification of viral vector genomes per diploid of each tissue was assayed by ddPCR (B). Fold-change in *Fluc* mRNA expression relative to *GAPDH* as assayed by qPCR with standard curve (C).

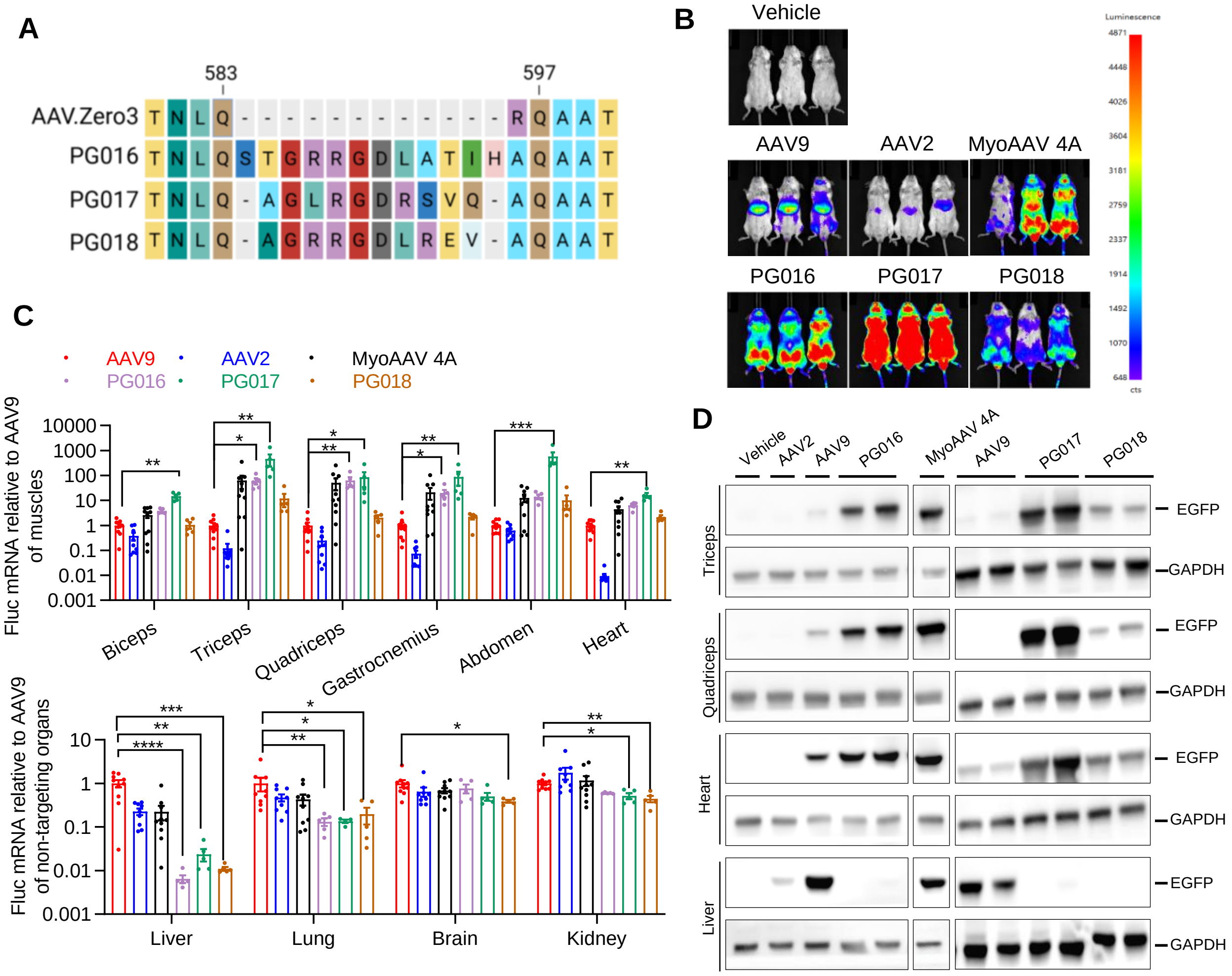
A
583
597
AAV.Zero3
PG016
PG017
PG018
B
Vehicle
AAV9
AAV2
MyoAAV 4A
PG016
PG017
PG018
Luminescence
C
AAV9
AAV2
MyoAAV 4A
PG016
PG017
PG018
Fluc mRNA relative to AAV9 of muscles
Biceps
Triceps
Quadriceps
Gastrocnemius
Abdomen
Heart
Fluc mRNA relative to AAV9 of non-targeting organs
Liver
Lung
Brain
Kidney
D
Vehicle
AAV2
AAV9
PG016
MyoAAV 4A
AAV9
PG017
PG018
Triceps
Quadriceps
Heart
Liver
EGFP
GAPDH

**Figure S4. Novel muscle targeting peptides based on low-background capsid exhibit selectively target to muscles with high efficiency**

A–D. 8-week-old BALB/c mice were systemically injected with $2 \times 10^{11}$ vg/mouse of AAV9-, AAV2-, MyoAAV 4A, PG016, PG017, or PG018-CAG-Fluc-P2A-EGFP and tissues were collected 21 days post-injection. Sequence alignments of the RGD-containing peptide insertion region of PG016, PG017, and PG018 compared with AAV.Zero3 (A). Representative whole body *in vivo* bioluminescence images (B), the images for the Vehicle, AAV9, and AAV2 control groups are the same as those presented in Figure 3A. These panels are derived from the same experimental batch and are reused here as a common internal control to enable a direct and consistent comparison with the novel capsids under investigation. Quantification of fold-difference in *Fluc* mRNA expression in various tissues (C) compared with normalized AAV9. Comparisons between AAV9 and each other group were performed using Kruskal-Wallis tests. Sample sizes were n = 10 for common internal control groups and n = 5 for test groups (PG016–PG018), with the following exceptions due to a single sample lacking a detectable signal: AAV2 in lung (n = 9) and PG017 in lung (n = 4). Each sample represents a biologically independent animal. Representative western blot images detecting EGFP and GAPDH in varied tissues (D).

**Table S1. Experimental design for barcode-mediated sorting of AAV variants in NHPs.**

Three monkeys received an intravenous co-injection of five AAV variants (MyoAAV 4A, PG016, PG017, PG018, AAV9) at a total dose of 3E13 GC/kg, equally distributed. Muscle (gastrocnemius, biceps, triceps, quadriceps) and liver punctures were performed at Day 14 and Day 28. Each muscle puncture sample was divided into three parts for analysis. Viral tropism was assessed via NGS barcode sequencing of the collected tissues. Data include raw DNA counts, calculated relative fold-enrichment versus AAV9, and summary statistics (mean ± STD) across biological replicates for each sample at Day 14 and Day 28.

**Table S2. NGS barcode sequencing counts and relative tissue tropism analysis for the five AAV variants in the liver of NHPs.**

**Table S3. NGS barcode sequencing counts and relative tissue tropism analysis for the five AAV variants in the gastrocnemius of NHPs.**

**Table S4. NGS barcode sequencing counts and relative tissue tropism analysis for the five AAV variants in the biceps of NHPs.**

**Table S5. NGS barcode sequencing counts and relative tissue tropism analysis for the five AAV variants in the triceps of NHPs.**

**Table S6. NGS barcode sequencing counts and relative tissue tropism analysis for the five AAV variants in the quadriceps of NHPs.**

**Table S7. AAV.eM demonstrates superior stability and reduced aggregation compared to MyoAAV 4A in AAV production.**

| Batch Scale (L) | Gene of Interest （GOI） | Capsid | Lysate Genome Titer (vg/mL) | DP Genome Titer (vg/mL) | Appearance | Z-average Diameter (nm) | PDI | SD (nm) |
|---|---|---|---|---|---|---|---|---|
| 200 | GOI-1 | MyoAAV 4A | 4.79E+11 | 1.08E+13 | Colorless Transparent | 785.66 | 0.383 | 30.85 |
| 200 | GOI-2 | MyoAAV 4A | 5.02E+11 | 1.41E+13 | Opalescent Cloudy | 1185.68 | 0.127 | 422.99 |
| | | | | 8.70E+12 | Colorless Transparent | 28.12 | 0.077 | 7.8 |
| | | | | 4.34E+12 | Colorless Transparent | 27.11 | 0.062 | 6.76 |
| 3 | GOI-3 | AAV.eM | 2.16E+11 | 3.01E+13 | Colorless Transparent | 30.33 | 0.062 | 7.56 |
| 3 | GOI-4 | AAV.eM | 3.22E+11 | 5.11E+13 | Colorless Transparent | 32.6 | 0.108 | 10.69 |
| 3 | GOI-5 | AAV.eM | 3.66E+11 | 1.18E+14 | Colorless Transparent | 29.37 | 0.047 | 6.37 |

Note： DP, drug product; PDI, polydispersity index; SD, standard deviation.

**Table S8. Primers**

| Primer | Sequence |
|---|---|
| **Backbone construction** | |
| Cap-F | CATCTTTGAACAATAAATGATTTAAATCAGGTATG |
| Cap-R | TCAACTGAAACGAATCAACCGGTTT |
| YJ69-R | TGTTGATTCTGTCCAGAACCGTTAATAGTCTTGCTCAAGTAATACAGGTACTG GTCGAT |
| YJ69-F | ACGGTTCTGGACAGAATCAACAAACGCTAAAATTTTCTCAGGCCGGAGCGAG T |
| YJ72-R | GTTGTTTTGAGTCACAGTGGTTGATACTCGCTGCTGGCGGTAACAG |
| YJ72-F | ATCAACCACTGTGACTCAAAACAACAACAGTGAATACTCGTGGACTGGAG |
| 247-R | TTGTCTGTTGCCAGCCTGGAGGTTGGTAGATACAGAACCATACT |
| 247-F | CCTCCAGGCTGGCAACAGACAAGCAGCTACCGCAGAT |
| 248-R | CTGCTTGTCTCTGGAGGTTGGTAGATACAGAACCATACT |
| 248-F | CAACCTCCAGAGACAAGCAGCTACCGCAGAT |
| **Peptide insertion** | |
| 249-R | TGTTGTCTGGTCTCCTCTTCCAGGTCCCTGGAGGTTGGTAGATACAGAACCAT ACT |
| 249-F | CCTGGAAGAGGAGACCAGACAACACTGAGACAAGCAGCTACCGCAGAT |
| 250-R | ATTGTTGAAATCGCCTCGTCTATTCTCCTGGAGGTTGGTAGATACAGAACCAT ACT |
| 250-F | AATAGACGAGGCGATTTCAACAATACCAGACAAGCAGCTACCGCAGAT |
| YJ107-R | GAGTTGTAGTCTCCTCTGCTGTTGCTCTGGAGGTTGGTAGATACAGAACCATA CT |
| YJ107-F | GCAACAGCAGAGGAGACTACAACTCCCTGAGACAAGCAGCTACCGCAGAT |
| **Viral titer** | |
| ITR-F | GGAACCCCTAGTGATGGAGTT |
| ITR-R | CGGCCTCAGTGAGCGA |
| **qPCR** | |
| Luciferase | F-AACCAGCGCCATTCTGATCA; R-TCGGGGTTGTTAACGTAGCC |
| GAPDH (mouse) | F-CAGGAGAGTGTTTCCTCGTCC; R-TTCCCATTCTCGGCCTTGAC |
| GAPDH (monkey) | F-GTCTCCTCTGACTTCAACAGCG; R-ACCACCCTGTTGCTGTAGCCAA |
| **ddPCR** | |
| Luciferase | F-GCTTCCACCTACCAGGCAT; R-TCTTACCGGTGTCCAAGTCC |
| | Probe-CCGCCAGGGCTACGGCCTGA |
| GAPDH (mouse) | F-GATGTCCTTGGTGCACACTC; R-GAGCTGAGATTGCCCCGC |
| | Probe-CCTCGTCCTTAAGTTCATAGTCTGTATTC |
| GAPDH (monkey) | F-CGGGTCTTTGCTGTCGTATG; R-CTGCACAGGGCGCCACAGCC |
| | Probe-GAGTAGGGACCTCCTGTCCT |
